# Expression and function of SLC38A5, an amino acid-coupled Na^+^/H^+^ exchanger, in triple-negative breast cancer and its relevance to macropinocytosis

**DOI:** 10.1042/BCJ20210585

**Published:** 2021-11-12

**Authors:** Sabarish Ramachandran, Souad R. Sennoune, Monica Sharma, Muthusamy Thangaraju, Varshini V. Suresh, Tyler Sneigowski, Yangzom D. Bhutia, Kevin Pruitt, Vadivel Ganapathy

**Affiliations:** 1 Department of Cell Biology and Biochemistry, Texas Tech University Health Sciences Center, Lubbock, TX 79430, U.S.A.; 2 Department of Immunology and Molecular Microbiology, Texas Tech University Health Sciences Center, Lubbock, TX 79430, U.S.A.; 3 Department of Biochemistry and Molecular Biology, Medical College of Georgia, Augusta University, Augusta, GA 30912, U.S.A.; 4 Department of Biology, Texas Tech University, Lubbock, TX 79409, U.S.A.

**Keywords:** breast cancer, cell proliferation, glutamine addiction, intracellular alkalinization, macropinocytosis, one-carbon metabolism

## Abstract

Metabolic reprogramming in cancer necessitates increased amino acid uptake, which is accomplished by up-regulation of specific amino acid transporters. However, not all tumors rely on any single amino acid transporter for this purpose. Here, we report on the differential up-regulation of the amino acid transporter SLC38A5 in triple-negative breast cancer (TNBC). The up-regulation is evident in TNBC tumors, conventional and patient-derived xenograft TNBC cell lines, and a mouse model of spontaneous TNBC mammary tumor. The up-regulation is confirmed by functional assays. SLC38A5 is an amino acid-dependent Na^+^/H^+^ exchanger which transports Na^+^ and amino acids into cells coupled with H^+^ efflux. Since cell-surface Na^+^/H^+^ exchanger is an established inducer of macropinocytosis, an endocytic process for cellular uptake of bulk fluid and its components, we examined the impact of SLC38A5 on macropinocytosis in TNBC cells. We found that the transport function of SLC38A5 is coupled to the induction of macropinocytosis. Surprisingly, the transport function of SLC38A5 is inhibited by amilorides, the well-known inhibitors of Na^+^/H^+^ exchanger. Down-regulation of SLC38A5 in TNBC cells attenuates serine-induced macropinocytosis and reduces cell proliferation significantly as assessed by multiple methods, but does not induce cell death. The Cancer Genome Atlas database corroborates SLC38A5 up-regulation in TNBC. This represents the first report on the selective expression of SLC38A5 in TNBC and its role as an inducer of macropinocytosis, thus revealing a novel, hitherto unsuspected, function for an amino acid transporter that goes beyond amino acid delivery but is still relevant to cancer cell nutrition and proliferation.

## Introduction

Cancer cells reprogramme their metabolism to best suit their need for accelerated proliferation associated with enhanced protein/lipid synthesis, DNA/RNA synthesis, and potentiated machinery for protection against oxidative stress and apoptosis [1–3]. This requires an increased supply of nutrients to feed into the altered metabolic pathways; amino acids represent an important group among such nutrients. Because of their hydrophilic nature, amino acids cannot permeate plasma membrane by diffusion; they enter the cells via selective transporters. There are more than three dozen amino acid transporters in mammalian cells [4]. They are grouped into seven different solute carrier gene families, based on the similarity in primary amino acid sequence. The increased demand for amino acids in cancer cells is met by the up-regulation of selective amino acid transporters. The most studied transporters for their role in cancer include SLC1A5 (alanine–serine–cysteine transporter 2 or ASCT2) [5–8], SLC7A5 (system L amino acid transporter 1 or LAT1) [5–9], SLC7A11 (system x−_c_ transporter or xCT) [5,6,10,11], and SLC6A14 (amino acid transporter B^0,+^ or ATB^0,+^) [5–8,12–14]. The contribution of each of these transporters to tumor promotion is the provision of selective amino acids to cancer cells, which might impact on a multitude of downstream metabolic pathways and signaling cascades.

SLC38 family contains several amino acid transporters [15], but there is little or no information in the literature on the role of these transporters in cancer. Only recently, a couple of studies have focused on SLC38A2 in breast cancer [16,17]. This transporter represents the classical Na^+^-coupled neutral amino acid transporter known as system A, cloned and functionally characterized in our laboratory [18]. It was called ‘system A’ because of its preference for the amino acid alanine. However, it transports not only alanine but also other small, hydrophilic, amino acids such as glycine and serine. Interestingly, glutamine is an excellent substrate for this transporter. SLC38A2 is induced by hypoxia and its expression is related to endocrine resistance in breast cancer [16]. In triple-negative breast cancer (TNBC), the level of SLC38A2 expression is associated with worse prognosis [17]. Both studies have implicated SLC38A2 in supplying glutamine to cancer cells. Notwithstanding these data suggesting a role for this transporter in breast cancer progression, its expression is actually down-regulated in breast cancer, particularly in TNBC (*P *< 1.5 × 10^−8^) (TCGA database). The decreased expression in cancer is paradoxical and counterintuitive, given the proposed role of the transporter as a tumor promoter.

In the present study, we focused on another member of the SLC38 family, namely SLC38A5 (also known as SNAT5—sodium-coupled neutral amino acid transporter 5). This transporter was also cloned in our laboratory [19,20]. It is referred to as SN2 transporter, meaning that it is the second isoform with functional features defined selectively for the amino acid transport system N. This system is Na^+^-coupled and accepts as substrates amino acids that contain nitrogen (N, hence the name system N) in the side chain (i.e. glutamine, asparagine, and histidine). However, functional characterization of the cloned transporter revealed two interesting features: (i) it also transports glycine, serine and methionine, with greater affinity towards serine and methionine, and (ii) it utilizes an outward-directed H^+^ gradient as a driving force in addition to an inward-directed Na^+^ gradient [19,20]. The transport mechanism involves the transfer of Na^+^ and amino acid substrate in one direction coupled to the transfer of H^+^ in the opposite direction, with the overall transport process being electroneutral. Stated differently, SLC38A5 is an amino acid-coupled Na^+^/H^+^ exchanger. The rationale for the present study to evaluate the expression and function of SLC38A5 in breast cancer is the following: (i) SLC38A5 is a transcriptional target for the oncogene c-Myc [21]; (ii) the substrate selectivity of SLC38A5 suggests a potentially key role for this transporter in cancer-associated metabolic pathways such as glutaminolysis (glutamine) and one-carbon metabolism (serine, glycine, and methionine) [14]; (iii) the efflux of H^+^ coupled to amino acid entry fulfills dual needs in cancer cells, namely supply of amino acids and removal of H^+^ [19,20], and (iv) Na^+^/H^+^ exchanger is known to promote macropinocytosis, a novel mechanism for cellular entry of nutrients from extracellular medium [22,23], suggesting a possibility that SLC38A5 as an amino acid-coupled Na^+^/H^+^ exchanger might also promote macropinocytosis in cancer cells.

## Materials and methods

### Materials

[2,3-^3^H]-l-Serine (specific radioactivity, >5 Ci/mmol) was purchased from Moravek, Inc. (Brea, CA, U.S.A.). [2-^3^H]-Glycine (specific radioactivity, 42.4 Ci/mmol) and [3,4-^3^H]-glutamine (specific radioactivity, 50.5 Ci/mmol) were purchased from PerkinElmer Corp. (Waltham, MA, U.S.A.). Ethylisopropyl amiloride (EIPA) and hexamethylene amiloride were from Sigma–Aldrich (St. Louis, MO, U.S.A.). Amiloride, benzyl amiloride and harmaline were from Cayman Chemical (Ann Arbor, MI, U.S.A.).

### Human tissues

Human breast cancer tissues and the surrounding normal tissues were obtained from the Augusta University Tumor Bank. The Tumor Bank collects and maintains a repository of de-identified tumor tissues and matched normal tissues; the tumor tissue collection has the approval from the Institutional Review Board and the Human Assurance Committee. These tissues are available to investigators without a separate approval from the Institutional Review Board.

### Animals

We used three different transgenic mouse models of spontaneous breast cancer for the analysis of Slc38a5 expression: MMTV-Neu (a model for HER2-positive breast cancer), MMTV-HRAS (a model for Ras activation-associated breast cancer), and MMTV-PyMT (polyoma middle T antigen-driven breast cancer, which starts initially as estrogen receptor positive and subsequently turns into estrogen receptor-negative). These mouse lines were originally obtained from the Jackson Laboratory (Bar Harbor, ME, U.S.A.): MMTV-Neu, stock no 002376; MMTV-HRAS, stock no. 004363; MMTV-PyMT, stock no. 002374). Since all mice used in the present study never went through pregnancy, we used mammary tissues from adult (∼12-week-old) virgin wild type mice as a control for comparison. These mice form spontaneous tumors in mammary glands at different ages: 8–10 months in MMTV-Neu and MMTV-HRAS mouse lines; 2–3 months in the MMTV-PyMT mouse line). The protocol was approved by the Institutional Animal Care and Use Committee of the Texas Tech University Health Sciences Center, Lubbock, TX, U.S.A. (IACUC approval number: 15002 for breeding protocol and 18005 for experimental protocol). All the experiments described in this study, including the animal experiments, were conducted at this institution. At the termination of the study, mice were sacrificed by cervical dislocation under CO_2_ anesthesia in accordance with the guidelines from the American Veterinary Medical Association.

### Cell lines and culture conditions

We used four human non-transformed mammary epithelial cell lines and seven estrogen receptor-negative breast cancer cell lines. The non-transformed cell lines are: HMEC (ATCC, Cat. no. PCS-600-010), HBL100 (ATCC, Cat. no. HTB-124), MCF10A (ATCC, Cat. no. CRL-10317), and MCF12A (ATCC, Cat. no. CRL10782). HMEC cells were cultured in Mammary Epithelial Cell Basal Medium (ATCC, Cat. no. PCS-600-030) and HBL100 cells were cultured in modified McCoy's 5a medium (ATCC, Cat. no. 30-2007). MCF10A and MCF12A cells were cultured in a special medium consisting of Dulbecco's modified Eagle's medium and Ham's F12 medium, in a 1 : 1 ratio, supplemented with 20 ng/ml human EGF, 0.01 mg/ml bovine insulin, 500 ng/ml hydrocortisone, and 100 ng/ml cholera toxin. All media contained 10% fetal bovine serum. Six of the seven estrogen receptor-negative cell lines were obtained from ATCC: BT20 (Cat. no. HTB-19), HCC1937 (Cat. no. CRL-2336), MDA-MB231 (Cat. no. CRM-HTB-26), MDA-MB436 (Cat. no. HTB-136), MDA-MB453 (Cat. no. HTB-131) and MDA-MB468 (Cat. no. HTB-132). SUM1315MO2 cell line was obtained from Expasy (Cat. no. CVCL_5589). BT20 cells were cultured in Eagle's Minimum Essential Medium (ATCC, Cat. no. 30-2003); HCC1937 cells were cultured in RPMI-1640 medium (ATCC, Cat. no. 30-2001); MDA-MB436 cells were cultured in Leibovitz's L-15 medium (ATCC, Cat. no. 30-2008), supplemented with 10 μg/ml insulin and 16 μg/ml glutathione. MDA-MB231, MDA-MB453, and MDA-MB468 cells were cultured in Leibovitz's L-15 medium. SUMO1315MO2 cells were cultured in Ham's F12 medium containing 10 ng/ml EGF and 5 μg/ml insulin. The estrogen receptor-positive cell line ZR-75.1 (ATCC, Cat. no. CRL-1500) was cultured in RPMI-1640 medium. All media contained 10% fetal bovine serum. All cell lines were mycoplasma-free. Three patient-derived xenograft (PDX) cell lines were provided by the TTUHSC Cancer Center; these are identified as TXBR-100, TXBR-237, and TXBR-247. These cell lines were cultured in the special medium described above for MCF10A.

### Uptake assays

Uptake of [^3^H]-serine was used to monitor the transport function of SLC38A5. Since SLC38A5 is a Na^+^-coupled transporter with the involvement of H^+^ movement in the opposite direction, the uptake assays were done using a pH 8.5 buffer to create an outwardly directly H^+^ gradient across the plasma membrane. As there are several amino acid transporters, even for serine that is used as the substrate in the present study, that are Na^+^-coupled, we cannot specifically monitor the function of SLC38A5 by using Na^+^-containing uptake buffer. However, unlike other Na^+^-coupled amino acid transporters, SLC38A5 is tolerant to Li^+^, meaning that this transporter functions when Na^+^ is replaced with Li^+^ [19,20]. Therefore, we used an uptake buffer with LiCl in place of NaCl. The composition of the uptake buffer was 25 mM Tris/Hepes, pH 8.5, containing 140 mM LiCl, 5.4 mM KCl, 1.8 mM CaCl_2_, 0.8 mM MgSO_4_, and 5 mM d-glucose. Serine is also a substrate for SLC7A5 (LAT1), which is a Na^+^-independent transporter; therefore, uptake via this transporter will contribute to the total uptake measured in the LiCl-containing buffer. To suppress serine uptake that might occur via SLC7A5, the uptake buffer contained 5 mM tryptophan to compete with and block the transport of serine mediated by SLC7A5; SLC38A5 does not transport tryptophan and therefore SLC38A5-mediated serine uptake is not affected by tryptophan. To determine the contribution of diffusion to the total uptake of serine, the same uptake buffer but with LiCl replaced isosmotically with *N*-methyl-d-glucamine chloride (NMDGCl) was used. Serine uptake was measured in two buffers: (i) LiCl-buffer, pH 8.5 with 5 mM tryptophan; (ii) NMDGCl-buffer, pH 8.5 with 5 mM tryptophan. The uptake in NMDGCl-buffer was subtracted from the uptake in LiCl-buffer to determine specifically the transport activity of SLC38A5.

Cells were seeded in 24-well culture plates (2 × 10^5^ cells/well) with the culture medium. On the day of uptake measurement, the culture plates were kept in a water bath at 37°C. The medium was aspirated and the cells were washed with uptake buffers. The uptake medium (250 μl) containing [^3^H]-serine as the tracer along with unlabeled serine (15 μM) was added to the cells. Following incubation for 15 min, the medium was removed and the cells were washed three times with ice-cold uptake buffer. The cells were then lysed in 1% sodium dodecyl sulfate/0.2 N NaOH and used for measurement of radioactivity. In the initial experiments for the identification of the transport function of SLC38A5, [^3^H]-glutamine and [^3^H]-glycine were also used as the substrates. But, for most of the experiments, [^3^H]-serine was used.

### Impact of intracellular acidification on SLC38A5 transport activity

Intracellular acidification in cultured cells was accomplished by the NH_4_Cl pretreatment as described previously [24]. Cells were cultured in 24-well culture plates. On the day of the uptake assay, cells were incubated with NaCl-buffer (same as the uptake buffer except that LiCl or NMDGCl was replaced with NaCl), pH 7.5 containing 25 mM NH_4_Cl for 30 min. The osmolality of the buffer was maintained by adjusting the concentration of NaCl (115 mM instead of 140 mM). Cells were then washed with the same buffer, but in the absence of NH_4_Cl (concentration of NaCl was increased to 140 mM), and then used to monitor the transport activity of SLC38A5 as described above. During the preincubation with NH_4_Cl, intracellular pH increases because of the conversion of NH_3_ to NH_4_^+^ inside the cells and then the pH comes down to physiological pH because of the various pH regulatory mechanisms. During the subsequent wash in the absence of NH_4_Cl, NH_4_^+^ within the cells dissociates to NH_3_ and H^+^, and NH_3_ diffuses out of the cell, leaving H^+^ inside with consequent intracellular acidification.

### RT-PCR

RNA was extracted from cells and mammary tissues from mice (normal and tumor) by TRIzol reagent (Thermo Fisher Scientific), and the RNA was reverse-transcribed using High-capacity cDNA reverse transcription kit (Thermo Fisher Scientific). PCR and quantitative PCR were performed with Takara Taq Hot Start Version (TaKaRa Biotechnology, Shiga, Japan) or Power SYBR Green PCR master mix (Thermo Fisher Scientific). Human-specific primers were used for cell lines whereas mouse-specific primers were used for mouse tissues. Primer sequences are shown in Supplementary Table S1. The relative mRNA expression was determined by the 2^−ΔΔ^Ct method. HPRT (hypoxanthine/guanine phosphoribosyl transferase) was used as a housekeeping gene for normalization.

### Macropinocytosis assay

Cells were plated on coverslips, placed in wells in a 12 well-plate, at a density of 1 × 10^5 ^cells/well, and cultured with 5% CO_2_ at 37°C using the culture media (with 10% fetal bovine serum) recommended for the respective cell lines. The cells were allowed to reach ∼70% confluency. About 16 h prior to macropinocytosis assay, the medium was removed and fresh culture medium without the fetal bovine serum was added. Cells were washed three times with buffers consisting of 140 mM NaCl, LiCl or NMDGCl buffer, pH 7.5, all of them containing the following as the common components: 5.4 mM KCl, 1.8 mM CaCl_2_, 0.8 mM MgSO_4_, and 5 mM d-glucose. Subsequently, the cells were exposed to the same respective buffers, but now containing TMR-dextran (100 μg/ml), with or without amino acids, and in the absence or presence of EIPA (100 μM) for 15 min at 37°C. Then, the cells were washed three times with the respective buffer alone and then fixed with 4% paraformaldehyde for 5 min, washed several times with phosphate-buffered saline, and mounted using Prolong diamond with 4,6-diamidino-2-phnenylindole (DAPI) as a nuclear marker. Cell images were taken using a Nikon confocal microscope, with a 60X objective and analyzed using the Nippon Ichi software. The images represent a maximum projection intensity derived from a Z-stack. The fluorescence quantification was done by measuring the corrected total cell fluorescence (CTCF) using Image J and the following formula:

CTCF =(integrated density)−(area of cell of interest)×(mean fluorescence of background).

For groups of cells (15–20 cells/field) in an image, an outline was drawn to measure integrated density, area of the cell of interest, and mean fluorescence of the adjacent background around the cell of interest. For each quantification, we randomly selected five separate fields, each containing a cluster of 15–20 cells and calculated CTCF for each field, and the resultant five values were averaged.

### shRNA-mediated down-regulation of SLC38A5

We used lentivirus-mediated transduction for shRNA-dependent down-regulation of SLC38A5 in MB231 cells. The shRNA constructs specific for human SLC38A5 were obtained from Sigma–Aldrich, which lists four constructs that had been validated for a significant silencing of the transporter (>65%) in the fibroblast cell line MCH58. We performed a preliminary study to screen the efficacy of all these four constructs for their ability to down-regulate SLC38A5 in MB231 cells. Based on this experiments, we selected two of these constructs (Cat. no. TRCN44005 and TRCN44007) that we found to down-regulate SLC38A5 in MB231 cells by 80%. The experimental protocol followed the manufacturer's directions for transduction and for the selection of stable clones. The extent of down-regulation in MB231 cells was monitored by measuring the SLC38A5 mRNA levels by quantitative RT-PCR and also by functional assays involving serine uptake.

### Cell proliferation assays

We used three different methods to monitor cell proliferation in control (transduced with an empty vector) and the two shRNA-transduced cell lines. First, we seeded 50 000 cells for each clone and cultured for 24 h and 48 h. At the end of these two time periods, we counted the cells. Second, we performed the colony-formation (clonogenic) assay with the three clones. Initial seeding was done with 500 cells/well and culture was continued for 7 days with culture medium replaced with fresh medium every other day. At the end of the 7-day time period, the medium was removed and the colonies were fixed with ice-cold methanol/acetone and then stained with Giemsa stain. Third, we monitored the incorporation of [^3^H]-thymidine into cellular DNA. Sub-confluent cultures were first established by seeding 5000 cells/well and culturing for 24 h. Then, the medium was replaced with fresh culture medium containing [^3^H]-thymidine (1 μCi) and the cells were cultured for 12 h, 24 h, and 48 h. At the end of these time periods, the medium was removed by aspiration and trichloroacetic acid (10% solution in water) was added to the wells to precipitate proteins and make the cellular macromolecules, including DNA, adhere to the culture dish. The wells were then washed with distilled water three times, and then the contents in the wells were solubilized with 1% sodium dodecyl sulfate/0.2 N NaOH and used for measurement of radioactivity.

### Cell cycle analysis

Cells were cultured to sub-confluency. They were then fixed in 50% ethanol, followed by treatment with 0.1% sodium citrate, 1 mg/ml RNase A, and 50 μg/ml propidium iodide, and then subjected to fluorescence-activated cell sorting (FACS) (Becton, Dickinson) analysis.

### Apoptosis assay

We used annexin V labeling to detect apoptotic cells and quantified them using flow cytometry. The Annexin V-FITC kit from BioLegend was used for this purpose. 7AAD was used as a fluorescent marker to label cellular DNA in necrotic cells where cells have undergone increased permeabilization due to cell death; annexin V was used to label phosphatidylserine in the outer leaflet of the plasma membrane in apoptotic cells. Flow cytometry of these cells allowed differential quantification of early apoptotic cells (annexin V negative, 7AAD negative), necrotic cells (annexin V positive, 7AAD positive), and viable cells (annexin V negative, 7AAD negative).

### Statistics

RT-PCR and uptake studies were done in triplicates and repeated twice. Statistical analysis was performed with a two-tailed, paired Student's *t*-test for single comparison and a *P*-value < 0.05 was considered statistically significant. Data are given as means ± S.E. For quantification of fluorescence signals in image analysis related to macropinocytosis and for analysis of inhibition of serine uptake by amiloride and its derivatives, ANOVA followed by Dunn's test was used to determine the significance of difference among the different groups.

## Results

### Differential expression of SLC6A14 and SLC38A5 in estrogen receptor-positive (ER+) breast cancer and triple-negative (TNBC) breast cancer

**Figure 1.**
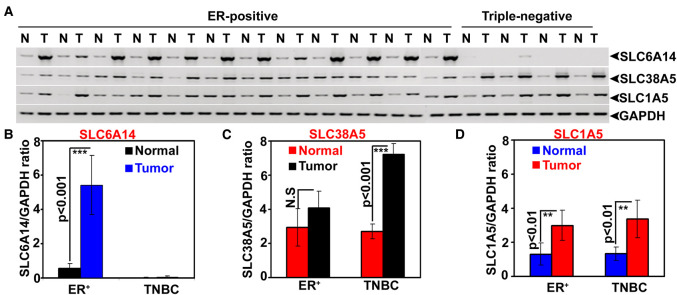
Relative expression of the amino acid transporters SLC6A14, SLC38A5, and SLC1A5 in paired tissues samples of breast cancer (ER+ breast cancer and ER-negative breast cancer) and normal mammary gland. (**A**) Semi-quantitative RT-PCR (N, normal mammary gland; T, tumor tissue). (**B**) Quantitative RT-PCR for SLC6A14. (**C**) Quantitative RT-PCR for SLC38A5. (**D**) Quantitative RT-PCR for SLC1A5. ER, estrogen receptor; TNBC, triple-negative breast cancer; GAPDH, glyceraldehyde-3-phosphate dehydrogenase.

We first examined primary tumor specimens from patients with ER+ breast cancer and TNBC for expression of SLC6A14 and SLC38A5. These were the same specimens used in a previously published study to examine the expression of SLC6A14 [25]. As reported earlier [25], SLC6A14 was expressed ER+ breast cancer but not in TNBC (Figure 1). In contrast, there was a marked up-regulation of SLC38A5 in TNBC (∼3-fold; *P *< 0.001; *n *= 4). There was a small increase in the expression of this transporter in ER+ breast cancer compared with corresponding adjacent normal tissues, but the difference was not statistically significant. We also studied the expression of SLC1A5. We found the expression of this transporter to be slightly increased in breast cancer but with no differential expression in ER+ breast cancer versus TNBC.

**Figure 2.**
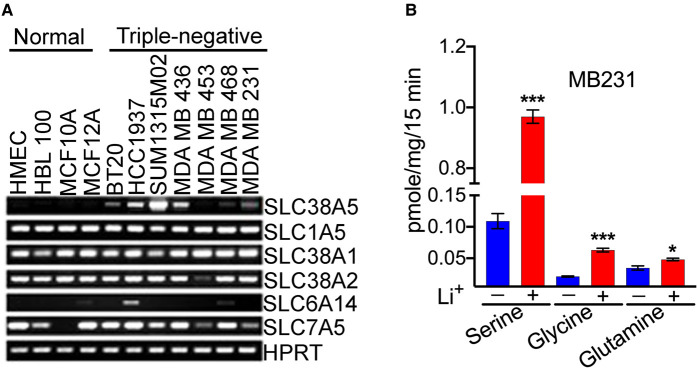
Differential expression of SLC38A5 and SLC6A14 in TNBC cell lines. (**A**) RT-PCR analysis of mRNAs for different amino acid transporters in four non-malignant breast epithelial cell lines and seven TNBC cell lines. HPRT, hypoxanthine/guanine phosphoribosyl transferase. (**B**) Transport activity of SLC38A5 in the TNBC cell line MB231 as monitored by the uptake of serine, glycine, and glutamine as the substrates for the transporter. The transport function of SLC38A5 was monitored in an uptake buffer (pH 8.5) containing 5 mM tryptophan to suppress the involvement of SLC7A5 in the uptake and by comparing the uptake in the presence and absence of Li^+^. The Li^+^-stimulatable uptake under these uptake conditions was taken as the transport activity specific for SLC38A5. *, *P *< 0.05; ***, *P *< 0.001, compared with uptake in the absence of Li^+^.

We then screened TNBC cell lines for the expression of various transporters that have glutamine as a common substrate. We focused on the transporters for glutamine because of its multiple biological functions in cancer cell proliferation and growth. For comparison, we used four non-malignant breast epithelial cell lines. Interestingly, the only amino acid transporter that was up-regulated in TNBC cell lines was SLC38A5 (Figure 2). There was no difference in the expression of SLC1A5 and SLC7A5 between non-malignant and TNBC cell lines. We also monitored the expression of two other members in the SLC38 family, namely SLC38A1 and SLC38A2, both of which accept glutamine as a substrate. Two recent studies have reported that SLC38A2 plays a critical role in supplying glutamine to breast cancer cells [16,17]. But, we did not find any difference in the expression of these two transporters between non-malignant and TNBC cell lines (Figure 2A).

### Functional evidence for SLC38A5 expression in TNBC cells

A unique feature that distinguishes SLC38A5 from other Na^+^-coupled amino acid transporters is its ability to function in the presence of Li^+^ in place of Na^+^. We have confirmed this feature of Li^+^ tolerance using cloned human SLC38A5 [19] and rat SLC38A5 [20]. As SLC38A5 recognizes glutamine, glycine, and serine as substrates [19,20], we examined the transport activity for these three amino acids in MB231 cells, a widely used prototypical TNBC cell line, in the presence and absence of Li^+^. The uptake buffer (pH 8.5) with Li^+^ was prepared by isosmotically replacing NaCl with LiCl. The buffer without Li^+^ was prepared by isosmotically replacing NaCl with NMDGCl. We found robust uptake of serine (0.4 μM) in these cells in LiCl-buffer (Figure 2B). The uptake in the absence of Li^+^ was only 10% of the total uptake measured in the presence of Li^+^. Glutamine uptake and glycine uptake were also increased in the presence of Li^+^, but the fold-stimulation elicited by Li^+^ was smaller compared with what was seen with serine uptake. Serine is one of the favored substrates for SLC38A5, and the most prominent Li^+^-dependent uptake for this amino acid confirms this substrate selectivity. We have confirmed the presence of SLC38A5 transport activity (i.e. Li^+^-stimulated uptake of serine, glycine, and glutamine) in two additional TNBC cell lines (HCC1937 and MB436) (Supplemental Figure S1).

**Figure 3.**
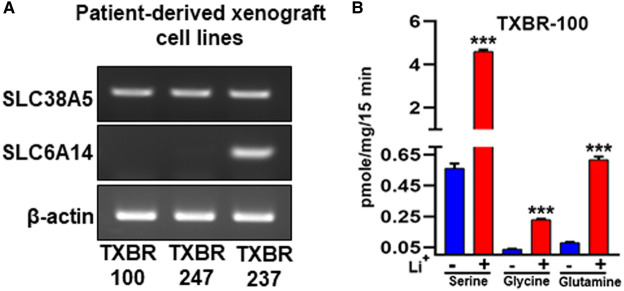
Expression and function of SLC38A5 in TNBC cell lines derived from patient-derived xenografts. (**A**) RT-PCR analysis of SLC38A5 and SLC6A14 mRNA expression in three cell lines. (**B**) Transport activity of SLC38A5 in the TNBC cell line TXBR-100 as monitored by the uptake of serine, glycine, and glutamine as the substrates for the transporter. The transport function of SLC38A5 was monitored in an uptake buffer (pH 8.5) containing 5 mM tryptophan to suppress the involvement of SLC7A5 in the uptake and by comparing the uptake in the presence and absence of Li^+^. The Li^+^-stimulatable uptake under these uptake conditions was taken as the transport activity specific for SLC38A5. ***, *P *< 0.001 compared with uptake in the absence of Li^+^.

We also used PDX TNBC cell lines for the analysis. We obtained three PDX cell lines from the Texas Cancer Cell Repository (www.TXCCR.org), which were developed from PDX samples representing TNBC. First, we monitored the expression of SLC6A14 and SLC38A5 in these cell lines and found all three cell lines to exhibit robust expression of SLC38A5 (Figure 3A). One of the three cell lines was positive for SLC6A14. We then selected one of the cell lines to monitor the transport function for SLC38A5. Li^+^-dependent serine (0.4 μM) uptake was robust in the TXBR-100 cell line (∼10-fold stimulation by Li^+^) (Figure 3B). Glycine uptake and glutamine uptake were also stimulated by Li^+^, but the fold-stimulation was the best for serine.

### Differential expression of Slc38A5 and Slc6a14 in three different mouse models of spontaneous breast cancer

**Figure 4.**
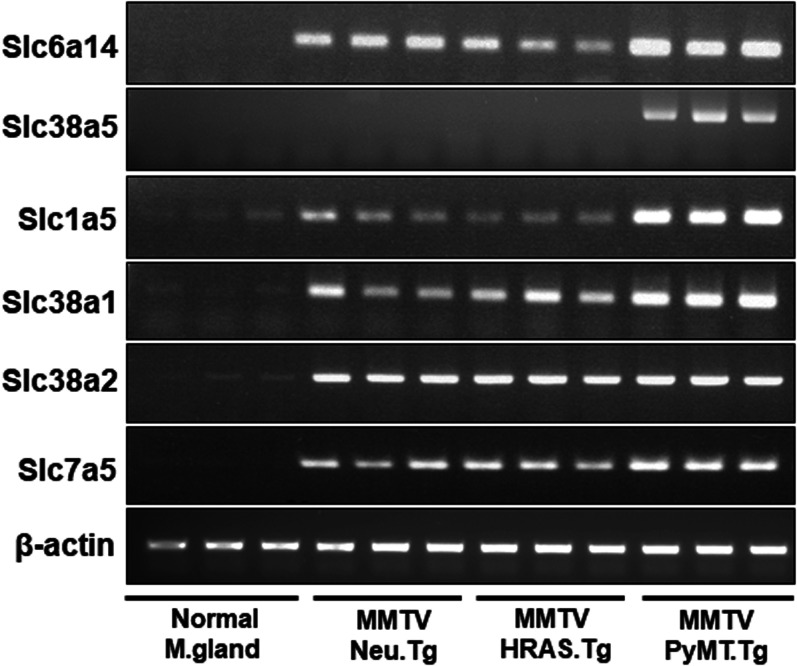
Differential expression of Slc38a5 in mouse models of spontaneous mammary tumors representative of different major subtypes. Tumors were collected from MMTV-Neu, MMTV-HRAS, and MMTV-PyMT female mice for RNA preparation. Mice used in the study did not go through breeding or pregnancy. Accordingly, virgin adult female wild type mice were used to collect mammary gland as the control. Since the age at which spontaneous tumors develop in the three mouse lines varies markedly (2–3 months of age for MMTV-PyMT mice whereas 8–10 months for the other two lines), we did not use age-matched wild type mice as controls. Instead, we used 3-month-old adult mice for this purpose. RNA was used for RT-PCR to monitor the expression pattern of several amino acid transporters that might be of relevance to tumor growth.

Transgenic mouse lines are available for studies of spontaneously occurring breast tumors of different major subclasses: MMTV-Neu transgenic mouse line develops HER-2 positive breast cancer, MMTV-HRAS transgenic mouse line develops oncogene (HRAS)-driven breast cancer, and MMTV-PyMT mouse line develops ER+ breast cancer initially which then transforms into ER-negative breast cancer [26]. A similar change in terms of the ER status occurs also in ∼30% of breast cancer in humans [27]. Normal virgin mammary tissue expresses neither Slc6a14 nor Slc38a5 (Figure 4). We found that Neu-driven tumors and HRAS-driven tumors express only Slc6a14. Slc38a5 expression was completely absent from these two subclasses. In contrast, in tumors driven by polyoma middle T tumor antigen (PyMT), the expression of both Slc6a14 and Slc38a5 was clearly evident. We also monitored the expression of the other members of the SLC38 family and of the other amino acid transporters whose expression has been shown to be up-regulated in breast cancer [6]. Slc38a1 and Slc38a2 are expressed at low levels in normal mammary tissue, and their expression is up-regulated in all three subclasses of breast cancer. The expression of Slc38a1 is slightly increased in PyMT-driven breast cancer than in Neu- and HRAS-driven breast cancers. In contrast, there is no difference in the expression of Slc38a2 among the three subclasses. Slc1a5 shows an expression pattern similar to that of Slc38a1; it is expressed at low levels in normal mammary tissue and it is up-regulated in all three subclasses of breast cancer. PyMT-driven breast cancer does show significantly higher expression of this transporter than in other breast cancer subtypes. Slc7a5 is expressed at negligible levels in normal mammary tissue and its expression is up-regulated in breast cancer; however, there is no difference in expression among the three subclasses of breast cancer.

### Functional features of Li^+^-coupled serine uptake in TNBC cell lines MB231 and TXBR-100

**Figure 5.**
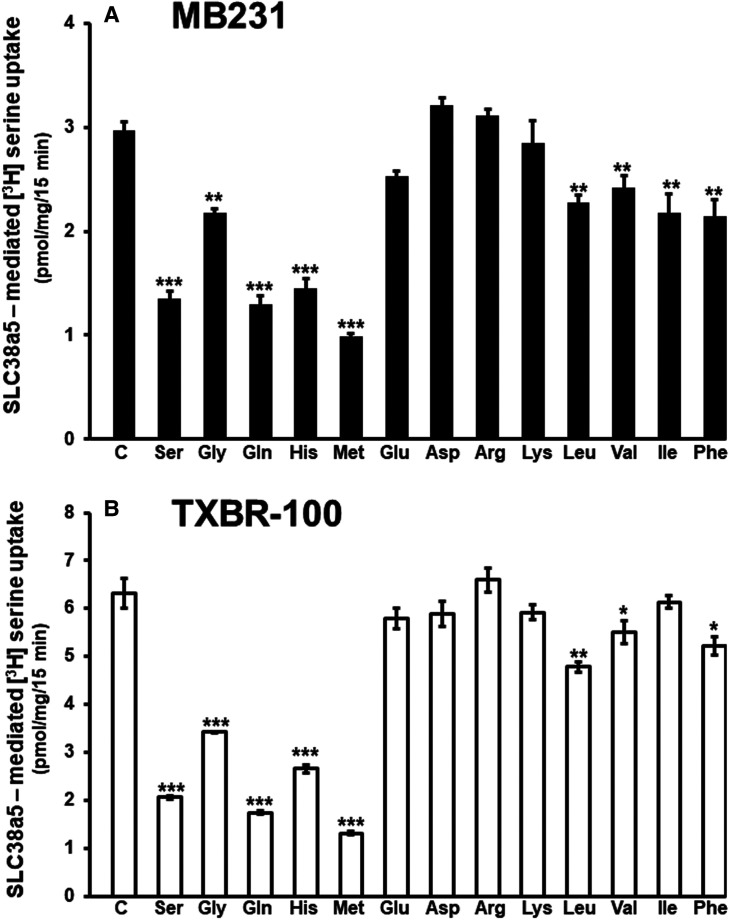
Substrate selectivity of Li^+^-coupled amino acid transport in TNBC cell lines. Uptake of [^3^H]-serine (15 μM) was measured in a LiCl-containing buffer, pH 8.5 (NaCl was replaced isosmotically with LiCl) in the absence or presence of various amino acids (5 mM). Uptake measured in the NMDGCl-buffer was subtracted from the uptake measured in the presence of LiCl (with or without the competing amino acids) to calculate the Li^+^-coupled uptake. In every case, the uptake buffer contained 5 mM tryptophan to suppress the contribution of serine uptake via SLC7A5. Data are means ± S.E (*n*=6). Statistical analysis was done using the paired Student's *t*-test by comparing the uptake in the presence and absence of unlabeled amino acids. (**A**) MB231 cells; (**B**) TXBR-100 cells. ***, *P *< 0.001 compared with control. C, control.

**Figure 6.**
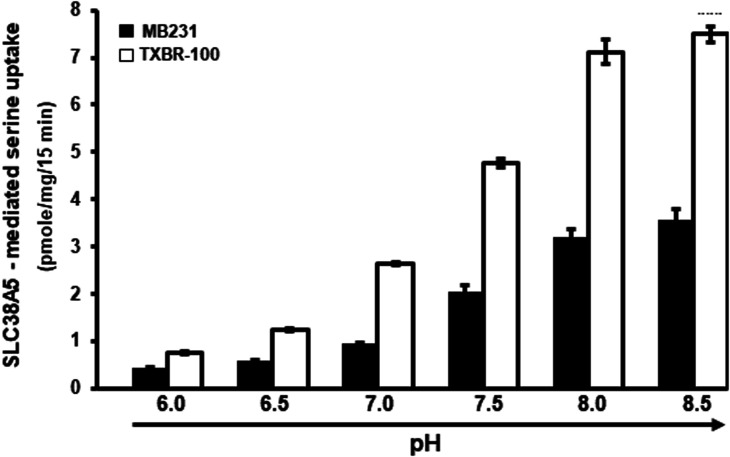
pH dependence of Li^+^-coupled [^3^H]-serine uptake (1 μM) in TNBC cell lines. Uptake buffer contained LiCl and 5 mM tryptophan. pH of the buffer was varied between 6.0 and 8.5. The pH 6.0 buffer was prepared using 25 mM Mes/Tris and the pH 8.5 buffer was prepared using 25 mM Tris/Hepes. The two buffers were mixed to prepare the buffers of other pH values. Serine uptake was measured in these buffers of different pH. Data are presented as means ± S.E (*n*=6). ′, MB231 cells; ≤, TXBR-100 cells.

SLC38A5 is not only Li^+^-tolerant but also selective for specific amino acids and its ability to mediate the influx of amino acids into cells is coupled to an outwardly directed H^+^ gradient across the plasma membrane. To confirm that the TNBC cells do express this particular transporter at the functional level, we studied the features of Li^+^-dependent serine uptake in MB231 and TXBR-100 cells. First, we examined the substrate selectivity by monitoring the ability of various amino acids to compete with [^3^H]-serine (15 μM) for the uptake process. At a concentration of 5 mM, unlabeled serine caused 60–70% inhibition of [^3^H]-serine uptake (Figure 5). The uptake was also inhibited by glutamine, histidine, glycine, and methionine. But, branched-chain amino acids (valine, isoleucine, and leucine) and aromatic amino acids (phenylalanine) were less effective as inhibitors of [^3^H]-serine uptake. Anionic amino acids (aspartate and glutamate) and cationic amino acids (arginine and lysine) had no significant effect. Analysis of saturation kinetics indicated participation of a single saturable process with a Michaelis constant of 2.3 ± 0.2 mM for serine (data not shown). We then studied the effect of extracellular pH on SLC38A5-mediated serine uptake to determine the potential role of a H^+^ gradient in the transport process. The uptake was low at acidic pH, but it increased as the extracellular pH was increased (Figure 6). As the extracellular pH is increased, the magnitude of the outwardly directed transmembrane H^+^ gradient across the plasma membrane increases proportionately. This suggests the potential involvement of the outward-directed H^+^ gradient as a driving force for the uptake process.

We also examined the impact of intracellular pH on the uptake process. For this, we used the NH_4_Cl prepulse method to cause intracellular acidification, another strategy to alter the transmembrane H^+^ gradient. We have used this method to demonstrate the energization of the peptide transporter PEPT1 [24] whose function is coupled to a transmembrane H^+^ gradient generated by Na^+^/H^+^ exchanger [28]. We found the uptake of serine (1 μM) in MB231 cells to be increased when intracellular pH was made acidic (control, 3.5 ± 0.2 pmoles/mg protein/15 min; NH_4_Cl prepulse, 4.7 ± 0.3 pmoles/mg protein/15 min; *P *< 0.01), amounting to ∼35% increase in uptake due to intracellular acidification. Serine uptake in TXBR-100 cells also responded to NH_4_Cl prepulse in a similar manner though the increase was a little bit lower (∼25%) but still statistically significant (*P* < 0.01).

### Functional coupling between SLC38A5-mediated amino acid uptake and macropinocytosis in TNBC cells

**Figure 7.**
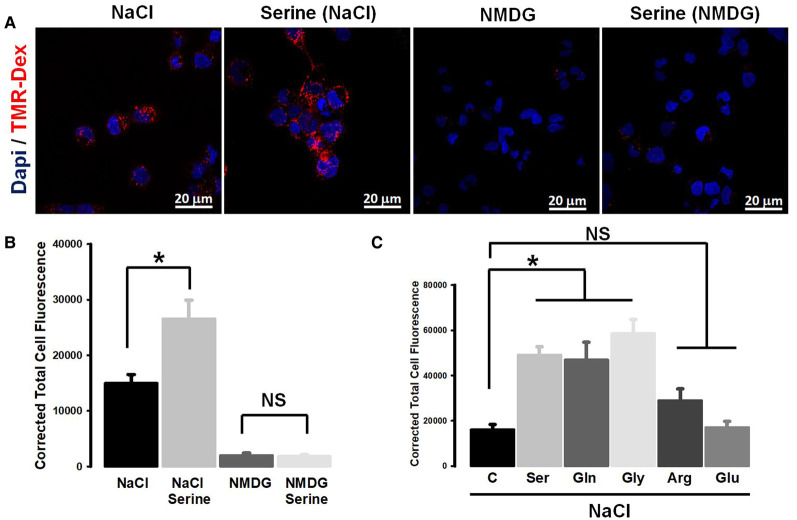
Promotion of macropinocytosis by SLC38A5-mediated transport function in the TNBC cell line TXBR-100. (**A**) Cells were incubated in buffers (pH 7.5) containing NaCl (Na^+^-buffer) or NMDGCl (Na^+^-free buffer) with and without serine (1 mM). All buffers contained TMR-dextran (100 μg/ml). The incubation was for 15 min at 37°C. The cells were then fixed with 4% paraformaldehyde for 5 min, washed with phosphate-buffered saline, and mounted using Prolong diamond with DAPI. The images of TMR-dextran uptake (red) and DAPI (blue) were taken using confocal microscopy with a 60X objective and analyzed using NIS software. The images represent a maximum projection intensity derived from a Z-stack. (**B**) Quantification of the fluorescence signals, measured as the corrected total cell fluorescence using Image J software. ANOVA followed by Dunn's test was used to determine the statistical significance among the groups. Data represent means ± S.E. *, *P* < 0.05. (**C**) Amino acid selectivity for the promotion of TMR-dextran uptake. Each amino acid was used at a concentration of 1 mM. Fluorescence signals were quantified and statistical significance analyzed as described above. *, *P *< 0.05; NS, not significant.

Based on the published reports that submembrane alkalinization on the cytoplasmic side of the plasma membrane caused by the activity of Na^+^/H^+^ exchanger promotes macropinocytosis [22,23], we wondered whether the function of SLC38A5 as an amino acid-dependent Na^+^/H^+^ exchanger might also induce macropinocytosis. To investigate this, we monitored the process of macropinocytosis in TXBR-100 cells by the cellular entry of the fluorescent marker TMR-dextran. When studied in the presence of a NaCl medium, the uptake of the fluorescent marker was markedly enhanced by serine, a substrate for SLC38A5 (Figure 7A,B). In contrast, serine did not induce the uptake of TMR-dextran in a Na^+^-free medium. In fact, the uptake of the marker was drastically decreased in the absence of Na^+^. When Na^+^ is removed from the medium, serine uptake does not occur via SLC38A5 because its transport activity is obligatorily coupled to Na^+^ (or Li^+^) and at the same time Na^+^/H^+^ exchanger functions in the opposite direction. Under normal conditions with Na^+^ present in the extracellular medium, the Na^+^/H^+^ exchanger mediates the entry of Na^+^ into cells coupled to the efflux of H^+^ from the cells, thus causing intracellular alkalinization. But, when Na^+^ is absent from the extracellular medium, the exchanger mediates the efflux of Na^+^ from the cells coupled to the influx of H^+^ into cells, causing intracellular acidification. Since macropinocytosis is promoted by alkalinization on the cytoplasmic surface of the plasma membrane, which alters actin polymerization and hence impacts the formation of macropinosomes, the decrease in the cellular uptake of the fluorescent marker for macropinocytosis is expected in a Na^+^-free extracellular medium.

If SLC38A5-mediated uptake of amino acids with its concomitant efflux of H^+^ was responsible for the serine-dependent increase in macropinocytosis in the NaCl-containing medium, this effect is expected to be restricted solely to the amino acid substrates of the transporter. To determine if this is true, we examined the influence of various amino acids on the uptake of the fluorescent marker. We found serine, glutamine, and glycine to be able to promote macropinocytosis (Figure 7C). In contrast, arginine and glutamate, which are not substrates for SLC38A5, failed to have any significant effect.

**Figure 8.**
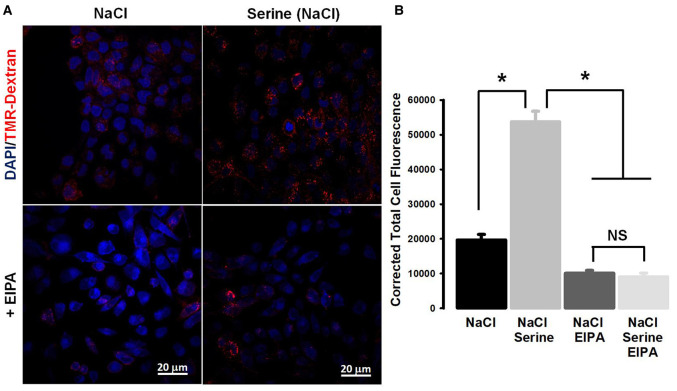
Blockade of SLC38A5-induced macropinocytosis by EIPA (ethylisopropyl amiloride). (**A**) Macropinocytosis was monitored using TMR-dextran in four different buffers (pH 7.5): NaCl, NaCl plus serine (1 mM), NaCl plus EIPA (100 μM) and NaCl plus serine (1 mM) and EIPA (100 μM). (**B**) Quantification of the fluorescence signals, and statistical significance among the groups. Data represent means ± S.E. *, *P *< 0.05; NS, not significant.

Amiloride and its derivatives are known inhibitors of macropinocytosis because of their ability to inhibit Na^+^/H^+^ exchanger [22,23]. Since SLC38A5 also functions as a Na^+^/H^+^ exchanger but in an amino acid-dependent manner, we wondered whether the promotion of macropinocytosis caused by serine in a NaCl-containing extracellular medium was sensitive to inhibition by amiloride. We addressed this by examining the effect of EIPA on Na^+^/serine-induced increase in the uptake of the fluorescent marker (Figure 8). EIPA blocked macropinocytosis almost completely under these conditions. EIPA inhibited the uptake of the fluorescent marker even in the absence of serine because it is an inhibitor of Na^+^/H^+^ exchanger. But, it was interesting that EIPA also blocked the serine-induced increase in the uptake of the marker, suggesting that this amiloride derivative might also be a blocker of the amino acid-dependent Na^+^/H^+^ activity of SLC38A5.

To further confirm the role of SLC38A5 as a promoter of macropinocytosis, we employed experimental conditions in which serine uptake occurs solely via SLC38A5. This was done by performing the experiments in the presence of 5 mM tryptophan, which blocks the uptake of serine via the amino acid transporter SLC7A5. Another change in the experimental condition was the replacement of NaCl with LiCl, thus restricting the uptake of serine solely to SLC38A5 without the participation of any other serine uptake system that might be coupled to Na^+^. We found that, in the presence of tryptophan, serine promoted macropinocytosis both NaCl medium and LiCl medium (Supplemental Figure S2A,B). Analysis of the data in Supplementary Figure S2 shows that tryptophan itself, which is not a substrate for SLC38A5, also induces macropinocytosis in a Na^+^-dependent manner (the signal is greater in NaCl than in LiCl), suggesting that some Na^+^-coupled transporter for tryptophan might also be involved in potentiating macropinocytis. However, there could be other explanations as well for this phenomenon because tryptophan metabolites are well known for their signaling functions. We also monitored serine-induced macropinocytosis in MB231 cells that have robust SLC38A5 expression and activity. When the experiment was performed in a NaCl-containing medium, serine-induced macropinocytosis in MB231 cells (Supplemental Figure S3). ZR 75.1, an ER+ cell line, expresses SLC38A5 at low levels (<25%) compared with the ER-negative cell lines MB231 and TXBR-100 (Supplemental Figure S4). Therefore, we monitored NaCl/serine-induced macropinocytosis in this cell line for comparison with the corresponding activity in MB231 cells. The SLC38A5-dependent macropinocytosis activity in ZR 75.1 cells was markedly less than in MB231 cells (Supplemental Figure S3).

Taken collectively, these experiments demonstrate that amino acid transport via SLC38A5 is coupled to the promotion of macropinocytosis in TNBC cells and that the amino acid-dependent Na^+^/H^+^ exchange activity of the transporter underlies this phenomenon.

### Inhibition of SLC38A5 transport function by amiloride and its derivatives

Amiloride and its derivatives are widely used as inhibitors of Na^+^/H^+^ exchangers. Since SLC38A5 functions as an amino acid-dependent Na^+^/H^+^ exchanger and it also induces macropinocytosis as the classical Na^+^/H^+^ exchanger does, we were curious to find out if amilorides would interact with SLC38A5 and interfere with its transport function. For this, we evaluated the effects of amiloride and its derivatives on SLC38A5-mediated serine transport in MB231 cells. The uptake was measured in a LiCl-buffer, pH 8.5, containing 5 mM tryptophan, the conditions that allow us to specifically monitor the SLC38A5-specific serine uptake. All four amilorides examined in the study inhibited SLC38A5 (Supplemental Figure S5). The most effective amilorides were EIPA and hexamethylene amiloride. Harmaline is known to interfere with Na^+^-coupled transport systems [29]. We found this compound also to have the ability to inhibit the transport function of SLC38A5 (Supplemental Figure S5).

### Attenuation of serine-induced macropinocytosis by shRNA-mediated silencing of SLC38A5

**Figure 9.**
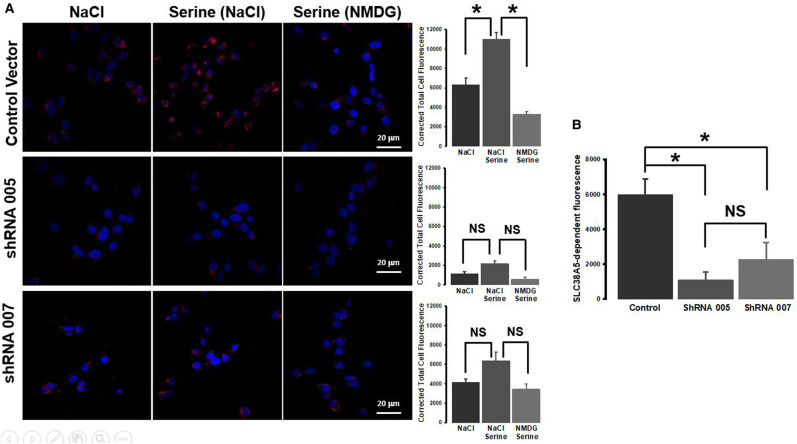
Attenuation of serine-induced macropinocytosis by SLC38A5 knockdown. SLC38A5 was silenced in MB231 cells with two different shRNAs. The experiments were done with stable cell clones: control (empty vector) and two shRNA clones. (**A**) Macropinocytosis was monitored with TMR-dextran in three different buffers (pH 7.5): NaCl, NaCl plus serine (1 mM), and NMDG (Na^+^-free) plus serine (1 mM). The fluorescent signals were quantified. *, *P *< 0.05; NS, not significant. (**B**) SLC38A5-specific macropinocytosis activity was calculated by subtracting the signals in NaCl from the signals in NaCl plus serine. *, *P *< 0.05; NS, not significant.

The experiments described thus far show that the amino acid-dependent Na^+^/H^+^ exchange activity of SLC38A5 induces macropinocytosis in TNBC cells. To further confirm this novel finding, we silenced SLC38A5 in MB231 cells with shRNA and then monitored serine-induced macropinocytosis. We found two shRNA constructs that worked the best in this particular cell line; both constructs reduced the levels of SLC38A5 mRNA by 80% (Supplemental Figure S6A). This decrease was associated with a 60–80% decrease in transport function as assessed by Li^+^-coupled serine uptake (Supplemental Figure S6B). We then used the control cells (empty vector) and the two shRNA-cells to monitor serine-induced macropinocytosis. In control cells, serine (1 mM) induced macropinocytosis in the presence of NaCl compared with an experimental condition where NaCl was replaced with NMDG chloride (Figure 9A). This serine-induced macropinocytosis was markedly reduced in the two shRNA-cells (Figure 9A), establishing the involvement of SLC38A5 in macropinocytosis. When only the Na^+^-stimulatable serine-dependent macropinocytosis (i.e. SLC38A5-dependent macropinocytosis) was considered, the activity was significantly reduced in both shRNA-cells (Figure 9B).

### Biological consequences due to loss of SLC38A5

**Figure 10.**
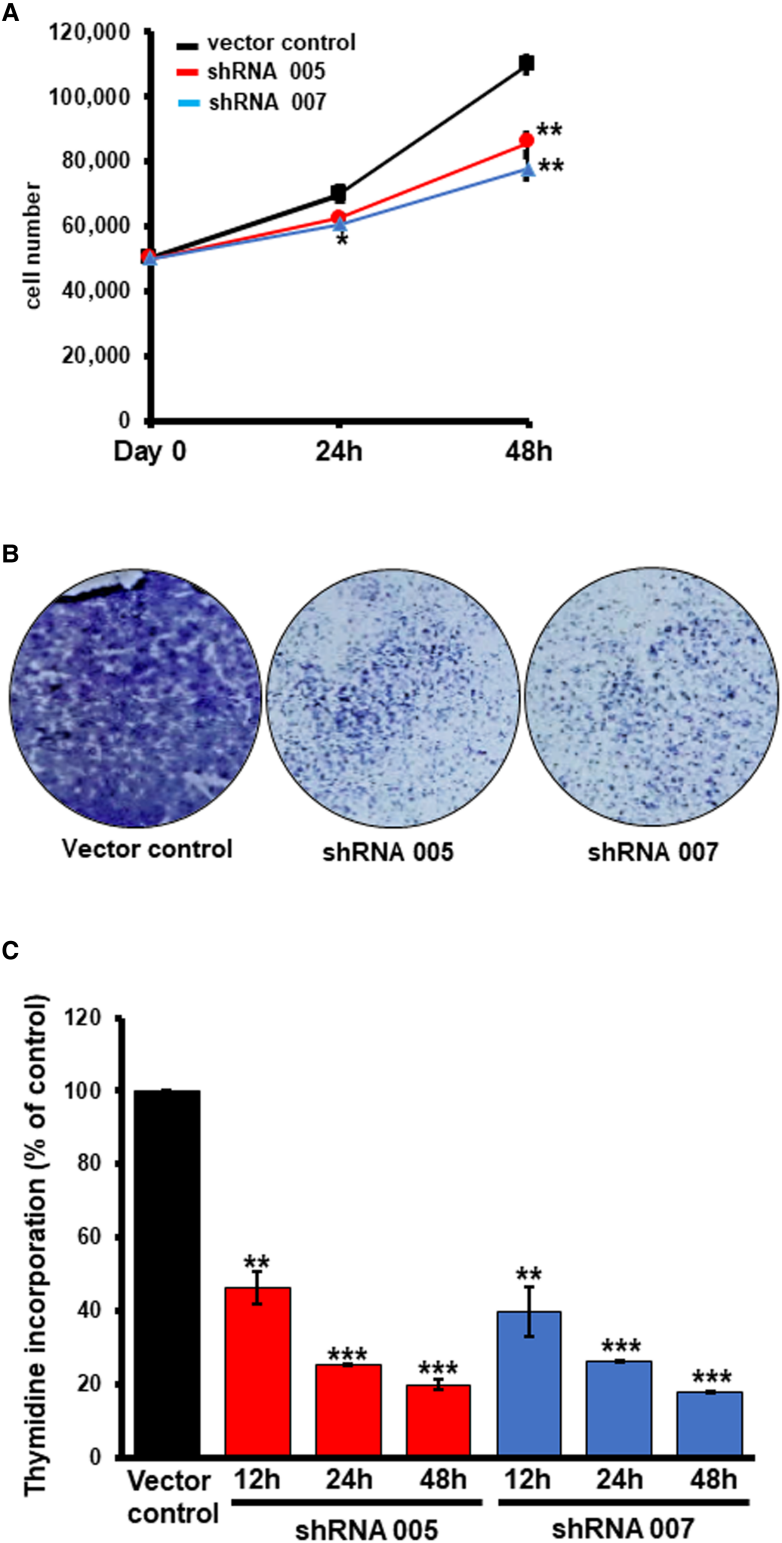
Decrease in cell proliferation due to SLC38A5 knockdown in MB231 cells. Cell proliferation in control (empty vector) and two shRNA-cells was monitored by three different assays. (**A**) Cell count. Cells were seeded in six-well culture plates (50 000 cells/well) and cultured for 24 h and 48 h. At the end of the culture period, cells were counted. Experiments were in triplicates. **, *P* < 0.05 compared with control cells. (**B**) Clonogenic assay. Representative images of the colonies stained with Giemsa stain are shown. The experiment was repeated three times with similar results. (**C**) Thymidine incorporation. Sub-confluent cultures were established by seeding 5000 cells/well and culturing for 24 h. Then, the medium was replaced with fresh culture medium containing [^3^H]-thymidine (1 μCi) and the cells were cultured for 12 h, 24 h, and 48 h. At the end of these time periods, the medium was removed by aspiration and trichloroacetic acid (10% solution in water) was added to precipitate cellular proteins and nucleic acids. The resultant sediments in the wells were washed to remove free [^3^H]-thymidine, and the radioactivity associated with the sediments was counted. **, *P *< 0.01 compared with control; ***, *P *< 0.001 compared with control.

**Figure 11.**
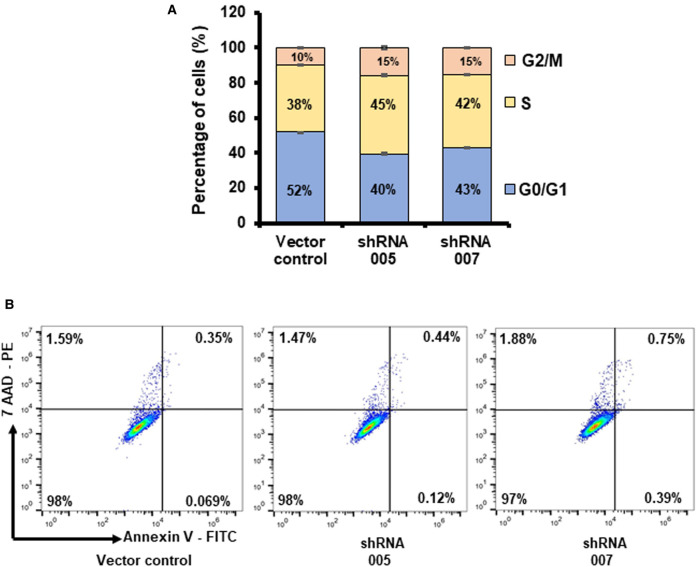
Analysis of cell cycle and apoptosis in control MB231 cells and shRNA-MB231 cells. (**A**) Cell cycle was monitored using propidium iodide and flow cytometry. (**B**) Apoptosis was monitored by annexin V labeling and cells were sorted by FACS.

As per our hypothesis, TNBC cells up-regulate SLC38A5 to meet their increased demand for amino acids such as glutamine, serine, and methionine. Our studies presented thus far here do show that SLC38A5 mediates the cellular entry of glutamine and serine; in addition, the transporter function is also associated with induction of macropinocytosis, which potentially facilitates cellular entry of proteins and other nutrients present in the extracellular fluid. If SLC38A5 does indeed provide amino acids (and other nutrients) to cancer cells, what would be the consequences in these cells due to loss of its function? To address this question, we monitored cell proliferation in control MB231 cells (empty vector) and in SLC38A5-silenced shRNA-MB231 cells. Three different assays (cell count, colony-formation, and thymidine incorporation) indicated that shRNA-induced SLC38A5 deficiency in these cells decreased cell proliferation; this was evident with both shRNAs (Figure 10). The magnitude of the decrease in cell proliferation ranged 50–80% depending on the assay. Cell cycle analysis showed that loss of SLC38A5 led to a decrease in the percent of cells in G0/G1 phase and an increase in the percent of cells in S and G2/M phases (Figure 11A). Interestingly and importantly, there was no cell death as assessed by annexin V labeling due to SLC38A5 deficiency (Figure 11B).

### Differential expression of SLC38A5 and SLC38A2 in different subtypes of breast cancer

The present investigation focuses on the expression of SLC38A5 in breast cancer. There have been two recent reports on the expression of SLC38A2, another member of the SLC38 family, in breast cancer [16,17]. SLC38A5 represents one of the subtypes of the amino acid transport system N, the other being SLC38A3; both function to mediate the influx of their amino acid substrates into cells coupled to Na^+^ symport and H^+^ antiport [19,20,30]. SLC38A2 is also a Na^+^-coupled amino acid transporter whose functional features resemble those of the amino acid transport system A [18], but there is no involvement of H^+^ in the transport mechanism associated with SLC38A2. SLC38A5 and SLC38A2 also differ in substrate specificity; the former possesses a much narrower amino acid selectivity whereas the latter is capable of transporting almost all neutral amino acids. We did not find any differential expression of SLC38A2 among the human breast cancer cells representative of ER+ or ER-negative breast cancer. A recently published report has shown that SLC38A2 expression is associated with oxidative stress resistance and poorer prognosis in TNBC [17]. Given that SLC38A5 as well as SLC38A2 might have relevance to breast cancer growth and progression, we analyzed the expression of these two transporters in breast cancer using the TCGA (The Cancer Genome Atlas) database. We found the expression of SLC38A5 to be significantly higher in primary breast tumor tissues than in normal breast tissue (Supplemental Figure S7A). The overexpression was evident in all three major subclasses of breast cancer (ER+ or luminal, HER2+, and basal or TNBC) (Supplemental Figure S7B). When the expression in ER+ and ER-negative breast cancer subtypes was analyzed separately, we found the expression to be much higher in ER-negative (TNBC) subtype than in ER+ (non-TNBC) subtype (Supplemental Figure S8). These observations corroborate the data from the present study. In contrast, the expression of SLC38A2 is reduced in primary breast cancer tissues compared with normal breast tissue (Supplemental Figure S9A). The decrease in expression was seen in all three major classes of breast cancer across the board; the most prominent down-regulation however was in TNBC (basal subtype) (Supplemental Figure S9B). Similarly, the expression of SLC38A3 was also found to be decreased in ER+, HER2+, and TNBC compared with normal mammary tissue (TCGA database).

## Discussion

The findings of the present study can be summarized as follows: (i) SLC6A14 and SLC38A5 are expressed differentially in ER+ breast cancer and TNBC; SLC6A14 is up-regulated in ER+ breast cancer whereas SLC38A5 is up-regulated in TNBC; (ii) the differential expression of these two transporters in different subtypes of breast cancer is evident also in cell lines representative of the two subtypes; (iii) there is no difference in the expression of other members of the SLC38 family between ER+ breast cancer and TNBC; (iv) the selective expression of SLC38A5 in TNBC is also observed in mouse models of spontaneous breast cancer for the different subtypes; (v) the transport function of SLC38A5 is demonstrable both in conventional TNBC cell lines and in PDX cell lines; (vi) SLC38A5 promotes macropinocytosis in TNBC cells; SLC38A5 is known to function as an amino acid-dependent Na^+^/H^+^ exchanger, a unique functional feature of the transporter, which seems to underlie the ability of SLC38A5 to promote macropinocytosis; (vii) the small molecule inhibitors of the classical Na^+^/H^+^ exchangers (members of the SLC9 family) also inhibit the transport function of SLC38A5, though not with the same potency; (viii) knockdown of SLC38A5 with shRNA markedly attenuated amino acid (serine)-induced macropinocytosis; (ix) shRNA-mediated silencing of SLC38A5 has significant biological consequences, decreasing cell proliferation but without having any noticeable effect on apoptotic cell death.

The Na^+^/Cl^−^-coupled broad-selective amino acid transporter SLC6A14 is expressed ER+ breast cancer cell lines but not in TNBC cell lines [25]. This is true also in primary breast tumor tissues [25]. SLC6A14 is a direct target for estrogen/ER, thus providing the molecular basis for its up-regulation in ER+ breast cancer [25]. Furthermore, the deletion of Slc6a14 in mice interferes with the growth of spontaneous mammary tumors [31]. These studies raised the question as to the identity of the transporter that is responsible for satisfying the amino acid needs in TNBC. Previous reports have shown that the amino acid transporters SLC1A5, SLC7A5, and SLC7A11 are up-regulated in TNBC [5-7]. But, the functional features of these three transporters are not ideally suited to meet the increased demands for amino acids in cancer cells [6]. SLC1A5 and SLC7A5 are obligatory amino acid exchangers, meaning that when these transporters mediate the influx of one of their amino acid substrates into cells, one of their other amino acid substrates is removed from the cells. Such a mechanism may be suitable for intracellular accumulation of specific amino acids for specialized intracellular functions as has been shown for leucine and its associated mTORC1 signaling [32]. But, this mechanism is not ideal for the general supply of amino acids to promote amino acid nutrition. SLC7A11 is also an exchanger with selectivity towards only cystine and glutamate; it transports cystine into cells coupled to the efflux of glutamate [33]. As such, this transporter is ideal to supply cysteine for glutathione synthesis and promote antioxidant machinery in cancer cells but not to meet the increased demands for amino acids. Therefore, we envisaged that there should be some other transporter(s) which is/are up-regulated in TNBC to supply amino acids in support of various metabolic pathways that are reprogrammed in cancer. We thought that SLC38A5 is an excellent candidate for TNBC because of its unique functional features [14,19,20]. It transports glutamine, glycine, serine, and methionine, coupled to the transmembrane Na^+^ gradient [19,20]. These amino acids are expected to maintain two important metabolic pathways that are accelerated in cancer, namely glutaminolysis associated with ATP production and lipid synthesis, and one-carbon metabolism associated with nucleotide synthesis and epigenetic control of gene expression [14]. When this transporter is silenced with shRNA, the cells undergo defective G0/G1 phase in the cell cycle; this could be due to the role of this transporter in amino acid supply for protein synthesis. There is also an indication of defective mitosis as evident from the increased percent of cells in S and G2/M phases as a result of SLC38A5 down-regulation. This could be at least partly due to defective DNA synthesis because serine plays an obligatory role in the one-carbon metabolism that is essential for nucleotide biosynthesis. However, these explanations are only hypothetical at present; additional work is needed to confirm or counter these explanations. The transporter is also coupled to H^+^ efflux, meaning that when amino acids are transported into cells via SLC38A5, the process is accompanied with the removal of H^+^ from the cells, thus leading to intracellular alkalinization [20]. Cancer cells would benefit from this process because intracellular alkalinization promotes mitogenesis [34] and macropinocytosis [22,23]. But, there have been no published reports investigating the expression of this amino acid transporter in breast cancer or any other cancers.

Whenever amino acid transporters in cancer are investigated, the sole focus is on the role of these transporters in the provision of amino acids to cancer cells. The present investigation departs from this norm; it focuses on the function of an amino acid transporter, which also possesses Na^+^/H^+^ exchange activity, and its relation to the endocytic process known as macropinocytosis. Since macropinocytosis facilitates the cellular entry of macromolecules, including proteins, present in the extracellular fluid, SLC38A5 contributes to amino acid nutrition in TNBC by two totally independent mechanisms, firstly by the traditional delivery of amino acids via the function of SLC38A5 as an amino acid transporter, and secondly by the promotion of macropinocytosis via the function of SLC38A5 as an amino acid-dependent Na^+^/H^+^ exchanger. This process of macropinocytosis delivers extracellular proteins to lysosomes with subsequent degradation of the proteins into amino acids for utilization by the cells.

The functional feature that is fundamental to the ability of SLC38A5 to promote macropinocytosis is its Na^+^/H^+^ exchange activity. SLC38A3 and SLC38A5 were once thought to be electrogenic transporters based on the inward currents induced by amino acid substrates in the presence of Na^+^ in frog oocytes that expressed these transporters heterologously [30]. But later studies showed that despite the fact that amino acids induce inward currents, this phenomenon is not related to amino acid transport *per se* [35,36]. Amino acid transfer into cells via SLC38A5 is electroneutral, with one Na^+^ going into the cells coupled to the efflux of H^+^. The inward current, seen when SLC38A5-expressing oocytes are exposed to amino acid substrates of the transporter, has nothing to do with the amino acid transfer but is related to amino acid-induced cation conductance. It is this cation conductance, not related to the entry of amino acids, that contributes to the inward currents. Whether this feature has any significance *in vivo* or whether it is a phenomenon that occurs only in the oocyte expression system needs to be investigated. The Na^+^/H^+^ exchange feature is unique only to SLC38A3 and SLC38A5. Schneider et al. [36] have discovered that SLC38A3 does not function only as an amino acid-dependent Na^+^/H^+^ exchanger; the transporter possesses Na^+^/H^+^ exchange activity that is not coupled to amino acid transport. In fact, the uncoupled Na^+^/H^+^ activity may be higher in magnitude than the activity coupled to amino acid transport. It is possible that a similar phenomenon also occurs with SLC38A5, which might have direct relevance to the transporter's ability to induce macropinocytosis. However, in the present study, we focused solely on the amino acid-dependent Na^+^/H^+^ exchange. There are about three dozen amino acid transporters in mammalian cells, and to the best of our knowledge, no amino acid transporter other than SLC38A3 and SLC38A5 possesses this interesting feature of Na^+^/H^+^ exchange. This is an important characteristic with direct relevance to cancer. When amino acids enter cancer cells via SLC38A5, it promotes the efflux of H^+^, which might contribute to the maintenance of intracellular pH. Cancer cells have an increased risk for intracellular acidification because of the production of lactic acid in aerobic glycolysis [3]. Here we have unraveled another interesting and important relationship between SLC38A5 and cancer cell biology. The amino acid-dependent Na^+^/H^+^ exchange activity of SLC38A5 promotes macropinocytosis, which may contribute significantly to amino acid nutrition in cancer cells. The fact that this transporter is selectively up-regulated in TNBC underscores its importance as a tumor promoter in TNBC. Even though SLC38A3 exhibits functional features that are almost identical with those of SLC38A5 [7], the expression of the former is actually decreased in breast cancer, and the decrease is independent of the hormone receptor status of the tumor (TCGA database). This suggests that SLC38A5 might have the potential as an actionable drug target for TNBC. At present, there are no targeted therapies available for TNBC and SLC38A5 might fill this void and offer a unique therapeutic target.

The pH dependence of SLC38A5 deserves some discussion with regard to the relevance of this transporter to cancer. Since the transporter function involves the movement of Na^+^ and amino acids in one direction coupled to the movement of H^+^ in the opposite direction, the influx of amino acids into cells in the presence of an inwardly directed Na^+^ gradient via the transporter is stimulated by an alkaline pH in the extracellular medium. The influx of amino acids is suppressed when the extracellular medium is acidic. In solid tumors such as the breast cancer, the tumor microenvironment is acidic, the conditions under which the influx of amino acids into cancer cells via the transporter would be low. It is possible that SLC38A5 plays a role in the transport of amino acids into cancer cells during the initial stages of carcinogenesis when the extracellular pH is not acidic. This is also the period when the function of the transporter would be associated with the promotion of macropinocytosis, thus providing additional mechanisms for the cancer cells to acquire nutrients. As the pH in the extracellular medium turns more acidic with time during tumor growth, the transporter might assume different functions. For example, the transporter might work in the reverse direction by facilitating the efflux of Na^+^ and amino acids from the cancer cells in exchange for extracellular H^+^. This potential reversal in the direction of movement in substrates will also depend on the relative magnitude of other amino acid transporters which function predominantly either as uniporters (e.g. SLC6A14 and SLC38A2) or obligatory exchangers (e.g. SLC1A5 and SLC7A5). In normal physiology, this potential for the bidirectional function of SLC38A3 and SLC38A5 is important in the glutamine–glutamate cycle that occurs in the brain between glutamatergic neurons and the surrounding astrocytes [37,38]. In this process, neurons release glutamate by exocytosis upon activation to elicit neurotransmission; then, the released glutamate is taken up by astrocytes via glutamate transporters to be converted into glutamine. This glutamine is then released into the extracellular fluid via SLC38A3 and SLC38A5, which is then taken up by neurons via SLC38A1 and SLC38A2 for subsequent conversion into glutamate for reuse as the neurotransmitter. In this scenario, SLC38A3 and SLC38A5 function in the reverse mode for the release of glutamine from the cells. SLC38A5 could carry out a similar function in solid tumors at advanced stages because of the acidic tumor microenvironment. Considering that glutamine, glycine, and cysteine are good substrates for SLC38A5, efflux of these amino acids via the transporter might result in decreased cellular levels of the antioxidant glutathione (γ-glutamyl-cysteinyl-glycine) in advanced tumors. This could potentially be relevant to the sensitivity and responsiveness of the tumors to selective chemotherapeutic agents whose clearance from the cells is dependent on the mercapturic pathway involving glutathione [39]. Tumors with high expression of SLC38A5 might be more sensitive to such drugs than tumors with low expression of SLC38A5; this is however only a hypothesis at present, but it is a concept that deserves further investigation in the future.

## Data Availability

All supporting data in relation to the studies reported here are provided in this manuscript.

## Open Access

Open access for this article was enabled by the participation of Texas Tech University Health Sciences Center in an all-inclusive *Read & Publish* pilot with Portland Press and the Biochemical Society under a transformative agreement with EBSCO.

## Abbreviations

CTCF: corrected total cell fluorescence
FACS: fluorescence-activated cell sorting
NMDGCl: *N*-methyl-d-glucamine chloride
PDX: patient-derived xenograft
PyMT: polyoma middle T tumor antigen
TNBC: triple-negative breast cancer

## References

[1] Pavlova, N.N. and Thompson, C.B. (2016) The emerging hallmarks of cancer metabolism. Cell Metab. 23, 27–47 10.1016/j.cmet.2015.12.006 26771115 PMC4715268

[2] Stine, Z.E., Walton, Z.E., Altman, B.J., Hsieh, A.L. and Dang, C.V. (2015) MYC, metabolism, and cancer. Cancer Discov. 5, 1024–1039 10.1158/2159-8290.CD-15-0507 26382145 PMC4592441

[3] Brown, T.P. and Ganapathy, V. (2020) Lactate/GPR1 signaling and proton motive force in cancer: role in angiogenesis, immune escape, nutrition, and Warburg phenomenon. Pharmacol. Ther. 206, 107451 10.1016/j.pharmthera.2019.107451 31836453

[4] Broer, S. and Fairweather, S.J. (2018) Amino acid transport across mammalian intestine. Compr. Physiol. 9, 343–373 10.1002/cphy.c170041 30549024

[5] Ganapathy, V., Thangaraju, M. and Prasad, P.D. (2009) Nutrient transporters in cancer: relevance to Warburg hypothesis and beyond. Pharmacol. Ther. 121, 29–40 10.1016/j.pharmthera.2008.09.005 18992769

[6] Bhutia, Y.D., Babu, E., Ramachandran, S. and Ganapathy, V. (2015) Amino acid transporters in cancer and their relevance to “glutamine addiction”: novel targets for the design of a new class of anticancer drugs. Cancer Res. 75, 1782–1788 10.1158/0008-5472.CAN-14-3745 25855379

[7] Bhutia, Y.D. and Ganapathy, V. (2016) Glutamine transporters in mammalian cells and their functions in physiology and cancer. Biochim. Biophys. Acta 1863, 2531–2539 10.1016/j.bbamcr.2015.12.017 26724577 PMC4919214

[8] Pochini, L., Scalise, M., Galluccio, M. and Indiveri, C. (2014) Membrane transporters for the special amino acid glutamine: structure/function relationships and relevance to human health. Front. Chem. 2, 61 10.3389/fchem.2014.00061 25157349 PMC4127817

[9] Cormerais, Y., Vucetic, M., Parks, S.K., and Pouyssegur, J. (2020) Amino acid transporters are a vital focal point in the control of mTORC1 signaling and cancer. Int. J. Mol. Sci. 22, 23 10.3390/ijms22010023 PMC7792758 33375025

[10] Lewerenz, J., Hewett, S.J., Huang, Y., Lambros, M., Gout, P.W., Kalivas, P.W., et al. (2013) The cystine/glutamate antiporter system x^-^_c_ in health and disease: from molecular mechanisms to novel therapeutic opportunities. Antioxid. Redox Signal. 18, 522–555 10.1089/ars.2011.4391 22667998 PMC3545354

[11] Lin, W., Wang, C., Liu, G., Bi, C., Wang, X., Zhou, Q. et al. (2020) SLC7A11/xCT in cancer: biological functions and therapeutic implications. Am. J. Cancer Res. 10, 3106–3126 https://www.ncbi.nlm.nih.gov/pmc/articles/PMC7642655 33163260 PMC7642655

[12] Sikder, M.O.F., Yang, S., Ganapathy, V. and Bhutia, Y.D. (2017) The Na^+^/Cl−-coupled, broad-specific, amino acid transporter SLC6A14 (ATB^0,+^): emerging roles in multiple diseases and therapeutic potential for treatment and diagnosis. AAPS J. 20, 12 10.1208/s12248-017-0164-7 29204754

[13] Nalecz, K.A. (2020) Amino acid transporter SLC6A14 (ATB^0,+^) – a target in combined anti-cancer therapy. Front. Cell Dev. Biol. 8, 594464 10.3389/fcell.2020.594464 33195271 PMC7609839

[14] Sneigowski, T., Korac, K., Bhutia, Y.D. and Ganapathy, V. (2021) SLC6A14 and SLC38A5 drive the glutaminolysis and serine-glycine-one-carbon pathways in cancer. Pharmaceuticals 14, 216 10.3390/ph14030216 33806675 PMC8000594

[15] Broer, S. (2014) The SLC38 family of sodium-amino acid co-transporters. Pflugers Arch. 466, 155–172 10.1007/s00424-013-1393-y 24193407

[16] Morotti, M., Bridges, E., Valli, A., Choudhry, H., Sheldon, H., Wigfield, S., et al. (2019) Hypoxia-induced switch in SNAT2/SLC38A2 regulation generates endocrine resistance in breast cancer. Proc. Natl Acad. Sci. U.S.A. 116, 12452–12461 10.1073/pnas.1818521116 31152137 PMC6589752

[17] Morotti, M., Zois, C.E., El-Ansari, R., Craze, M.L., Rakha, E.A., Fan, S.J., et al. (2021) Increased expression of glutamine transporter SNAT2/SLC38A2 promotes glutamine dependence and oxidative stress resistance, and is associated with worse prognosis in triple-negative breast cancer. Br. J. Cancer 124, 494–505 10.1038/s41416-020-01113-y 33028955 PMC7852531

[18] Sugawara, M., Nakanishi, T., Fei, Y.J., Huang, W., Ganapathy, M.E., Leibach, F.H. et al. (2000) Cloning of an amino acid transporter with functional characteristics and tissue expression pattern identical to that of system A. J. Biol. Chem. 275, 16473–16477 10.1074/jbc.C000205200 10747860

[19] Nakanishi, T., Sugawara, M., Huang, W., Martindale, R.G., Leibach, F.H., Ganapathy, M.E. et al. (2001) Structure, function, and tissue expression pattern of human SN2, a subtype of the amino acid transport system N. Biochem. Biophys. Res. Commun. 281, 1343–1348 10.1006/bbrc.2001.4504 11243884

[20] Nakanishi, T., Kekuda, R., Fei, Y.J., Hatanaka, T., Sugawara, M., Martindale, R.G. et al. (2001) Cloning and functional characterization of a new subtype of the amino acid transport system N. Am. J. Physiol. Cell Physiol. 281, C1757–C1768 10.1152/ajpcell.2001.281.6.C1757 11698233

[21] Wise, D.R., DeBerardinis, R.J., Mancuso, A., Sayed, N., Zhang, X.Y., Pfeiffer, H.K. et al. (2008) Myc regulates a transcriptional program that stimulates mitochondrial glutaminolysis and elads to glutamine addiction. Proc. Natl Acad. Sci. U.S.A. 105, 18782–18787 10.1073/pnas.0810199105 19033189 PMC2596212

[22] Commisso, C. (2019) The pervasiveness of macropinocytosis in oncological malignancies. Phil. Trans. R. Soc. Lond. B Biol. Sci. 374, 20180153 10.1098/rstb.2018.0153 30967003 PMC6304744

[23] Stow, J.L., Hung, Y. and Wall, A.A. (2020) Macropinocytosis: insights from immunology and cancer. Curr. Opin. Cell Biol. 65, 131–140 10.1016/j.ceb.2020.06.005 32745890

[24] Brandsch, M., Brandsch, C., Prasad, P.D., Ganapathy, V., Hopfer, U. and Leibach, F.H. (1995) Identification of a renal cell line that constitutively expresses the kidney-specific high-affinity H^+^/peptide cotransporter. FASEB J. 9, 1489–1496 10.1096/fasebj.9.14.7589991 7589991

[25] Karunakaran, S., Ramachandran, S., Coothankandaswamy, V., Elangovan, S., Babu, E., Periyasamy-Thandavan, S. et al. (2011) SLC6A14 (ATB^0,+^) protein, a highly concentrative and broad specific amino acid transporter, is a novel and effective drug target for treatment of estrogen receptor-positive breast cancer. J. Biol. Chem. 286, 31830–31838 10.1074/jbc.M111.229518 21771784 PMC3173074

[26] Lin, E.Y., Jones, J.G., Li, P., Zhu, L., Whitney, K.D., Muller, W.J. et al. (2003) Progression to malignancy in the polyoma middle T oncoprotein mouse breast cancer model provides a reliable model for human diseases. Am. J. Pathol. 163, 2113–2126 10.1016/S0002-9440(10)63568-7 14578209 PMC1892434

[27] Lapidus, R.G., Nass, S.J. and Davidson, N.E. (1998) The loss of estrogen and progesterone receptor gene expression in human breast cancer. J. Mammary Gland Biol. Neoplasia 3, 85–94 10.1023/A:1018778403001 10819507

[28] Kennedy, D.J., Leibach, F.H., Ganapathy, V. and Thwaites, D.T. (2002) Optimal absorptive transport of the dipeptide glycylsarcosine is dependent on functional Na^+^/H^+^ exchange activity. Pflugers Arch. 445, 139–146 10.1007/s00424-002-0910-1 12397398

[29] Alvarado, F., Brot-Laroche, E., L'Herminier, M., Murer, H. and Stange, G. (1979) The effect of harmaline on intestinal sodium-transport and on sodium-dependent D-glucose transport in brush-border membrane vesicles from rabbit jejunum. Pflugers Arch. 382, 35–41 10.1007/BF00585901 574938

[30] Fei, Y.J., Sugawara, M., Nakanishi, T., Huang, W., Wang, H., Prasad, P.D. et al. (2000) Primary structure, genomic organization, and functional and electrogenic characteristics of human system N1, a Na^+^- and H^+^-coupled glutamine transporter. J. Biol. Chem. 275, 23707–23717 10.1074/jbc.M002282200 10823827

[31] Babu, E., Bhutia, Y.D., Ramachandran, S., Gnanaprakasam, J.P., Prasad, P.D., Thangaraju, M. et al. (2015) Deletion of the amino acid transporter Slc6a14 suppresses tumour growth in spontaneous mouse models of breast cancer. Biochem. J. 469, 17–23 10.1042/BJ20150437 26173258

[32] Nicklin, P., Bergman, P., Zhang, B., Triantafellow, E., Wang, H., Nyfeler, B. et al. (2009) Bidirectional transport of amino acids regulates mTOR and autophagy. Cell 136, 521–534 10.1016/j.cell.2008.11.044 19203585 PMC3733119

[33] Bridges, C.C., Kekuda, R., Wang, H., Prasad, P.D., Mehta, P., Huang, W. et al. (2001) Structure, function, and regulation of human cystine/glutamate transporter in retinal pigment epithelial cells. Invest. Ophthalmol. Vis. Sci. 42, 47–54 https://iovs.arvojournals.org/article.aspx?articleid=2162479 11133847

[34] Flinck, M., Kramer, S.H. and Pedersen, S.F. (2018) Roles of pH in control of cell proliferation. Acta Physiol. (Oxford) 223, e13068 10.1111/apha.13068 29575508

[35] Broer, A., Albers, A., Setiawan, I., Edwards, R.H., Chaudhry, F.A., Lang, F. et al. (2002) Regulation of the glutamine transporter SN1 by extracellular pH and intracellular sodium ions. J. Physiol. 539, 3–14 10.1113/jphysiol.2001.013303 11850497 PMC2290136

[36] Schneider, H.P., Broer, S., Broer, A. and Deitmer, J.W. (2007) Heterologous expression of the glutamine transporter SNAT3 in *Xenopus* oocytes is associated with four modes of uncoupled transport. J. Biol. Chem. 282, 3788–3798 10.1074/jbc.M609452200 17148440

[37] Eid, T., Gruenbaum, S.E., Dhaher, R., Lee, T.S.W., Zhou, Y. and Danbolt, N.C. (2016) The glutamate-glutamine cycle in epilepsy. Adv. Neurobiol. 13, 351–400 10.1007/978-3-319-45096-4_14 27885637

[38] Jenstad, M. and Chaudhry, F.A. (2013) The amino acid transporters of the glutamate/GABA-glutamine cycle and their impact on insulin and glucagon secretion. Front. Endocrinol. 4, 199 10.3389/fendo.2013.00199 PMC3876026 24427154

[39] Yadav, S., Zajac, E., Singhal, S.S. and Awasthi, S. (2007) Linking stress-signaling, glutathione metabolism, and signaling pathways and xenobiotic transporters. Cancer Metastasis Rev. 26, 59–69 10.1007/s10555-007-9043-5 17260165

