# Supplementary material for: Expression and function of SLC38A5, an amino acid-coupled Na+/H+ exchanger, in triple-negative breast cancer and its relevance to macropinocytosis
Source: Biochem J. 2021 Nov 12;478(21):3957–76. doi: 10.1042/BCJ20210585 (PMC8652584; doi:10.1042/BCJ20210585)
Supplement: Supplementary Figures S1-S9 and Table S1 [file BCJ-478-3957-s1.pdf]

Supplementary Material

Supplementary Table 1. PCR primer sequences

Human-specific primers

Gene	Forward Primer	Reverse Primer
**SLC6A14**	ATC GTC TGG CAA GGT GGT AT	TGA GTG GCA GCA TCT TTC CAT
**SLC38A5**	GTT GGG GCC ATG TCC AGT TA	AGT GTT TCA TGA GGG CGA GG
**SLC1A5**	GAG ACT CCA AGG GGC TCG C	CAC AAG CAG GTT GGC TCG AAG
**SLC38A1**	TTT GGA GTC GTA GGA GTT ACA TCT	TGG AAA CTG GAG GAA GAG AAA GA
**SLC38A2**	GCA GTG GAA TCC TTG GGC TT	ATA AAG ACC CTC CTT CAT TGG CA
**SLC7A5**	CGC TCT TCC CCA CCT GC	GAC ACA TCA CCC TTC CCG AT
**GAPDH**	CCA CTC CTC CAC CTT TGA C	ACC CTG TTG CTG TAG CCA
**HPRT**	GCG TCG TGA TTA GCG ATG ATG AAC	CCT CCC ATC TCC TTC ATG ACA TCT

Mouse-specific primers

Gene	Forward Primer	Reverse Primer
**Slc6a14**	CCA ATG GCG GAG GTG CTT TC	ATC AGG ACC ATC GTG ATT CCC
**Slc38a5**	CAC AAC GTT GGG GCT ATG TC	TGC TTG TGT ACC CCA GGT AG
**Slc1a5**	TTC CCC TCC AAT CTG GTG TCT	CCA CCT CAC AGA GAA GCT GGA C
**Slc38a1**	ACG CGT GCA CAC CAA AGT AT	AAA GAT GGC CGT CAG GAA GT
**Slc38a2**	TTG CAG GCC ACG CTA TTT CA	AGC ACA GCC AAT CGG ACA ACA A
**Slc7a5**	GCT GAC GAA CCT GGC CTA TT	TAG TTC CCG AAG TCC ACA GC
**β-actin**	CTG GCA CCA CAC CTT CTA	GGG CAC AGT GTG GGT GAC

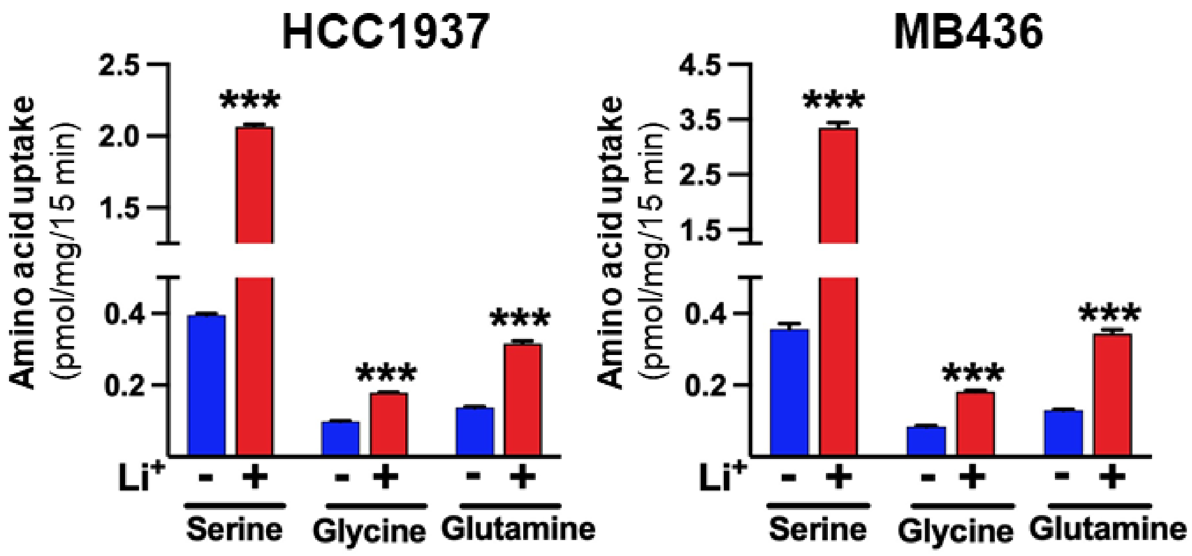

Fig. S1. Transport activity of SLC38A5 in the TNBC cell lines HCC1937 and MB436 as monitored by the uptake of serine, glycine, and glutamine as the substrates for the transporter. The transport function of SLC38A5 was monitored in an uptake buffer (pH 8.5) containing 5 mM tryptophan to suppress the involvement of SLC7A5 in the uptake and by comparing the uptake in the presence and absence of Li^+. The Li^+-stimulatable uptake under these uptake conditions was taken as the transport activity specific for SLC38A5. ***, $p<0.001$ compared to uptake in the absence of Li^+.

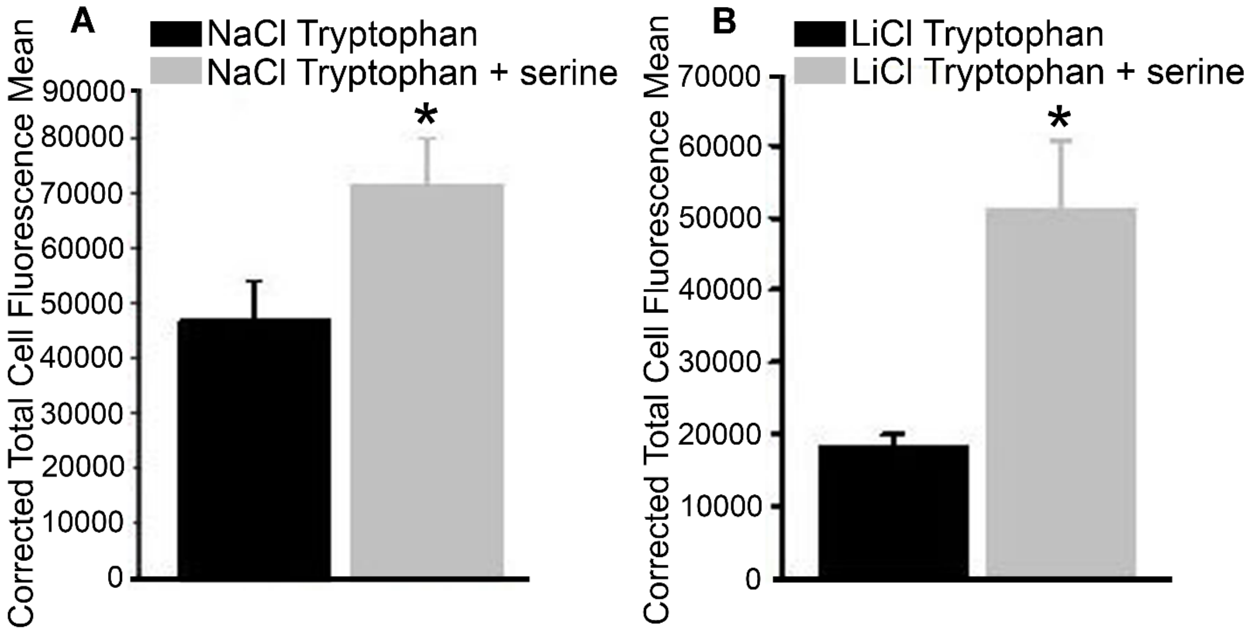

Fig. S2. Serine-induced macropinocytosis in the presence of Na^+ (A) or Li^+ (B) in the TNBC cell line TXBR-100. Macropinocytosis was monitored by the uptake of TMR-dextran by measuring intracellular fluorescence. Cells were incubated with TMR-dextran in NaCl-buffer (pH 7.5) or LiCl-buffer (pH 7.5) containing 5 mM tryptophan with and without serine (1 mM). Fluorescence signals were quantified and reported as Corrected Total Cell Flourescence (means ± S. E.).

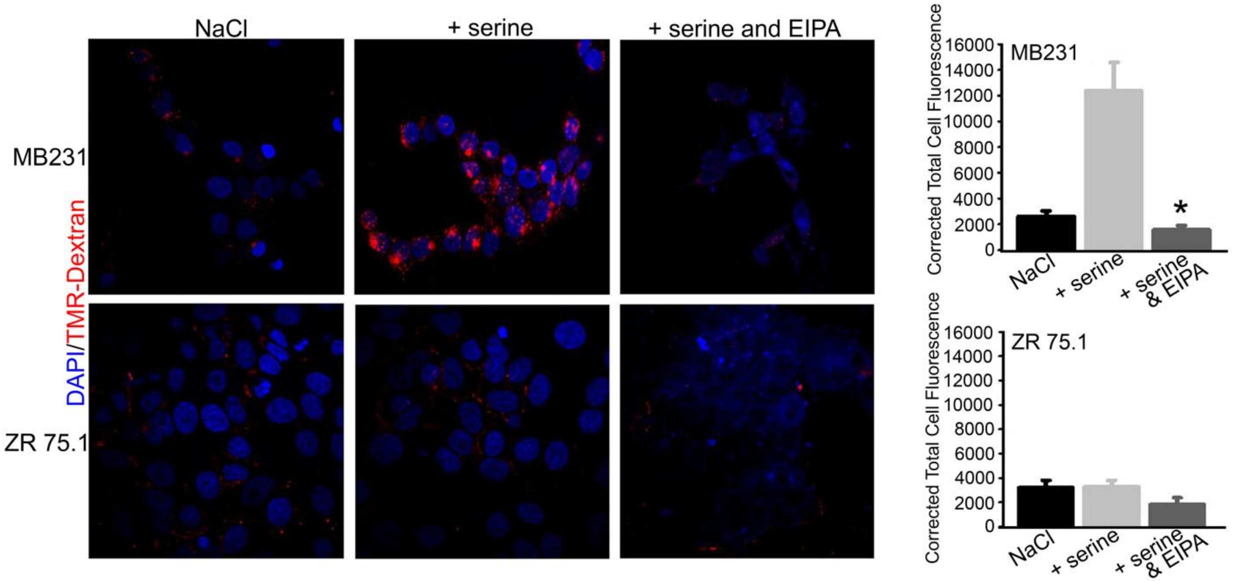

Fig. S3. Differential influence of serine on macropinocytosis in the TNBC cell line MB231 and the ER+ cell line ZR 75.1. Macropinocytosis was monitored using TMR-dextran in NaCl-buffer with and without serine (1 mM) and with and without EIPA (100 μM). The fluorescence signals were quantified and reported as Corrected Total Cell Fluorescence (means ± S.E.).

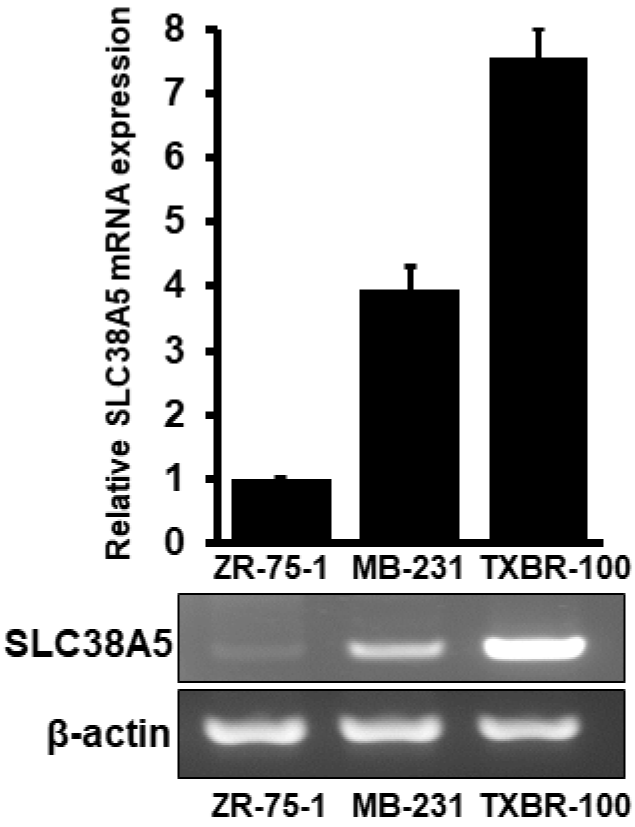

Fig. S4. Relative expression of SLC38A5 mRNA in the ER-positive human breast cancer cell line ZR-75-1 compared to two ER-negative human breast cancer cell lines (MB-231 and TXBR-100). Data are from RT-qPCR and β-actin mRNA was used as the internal control.

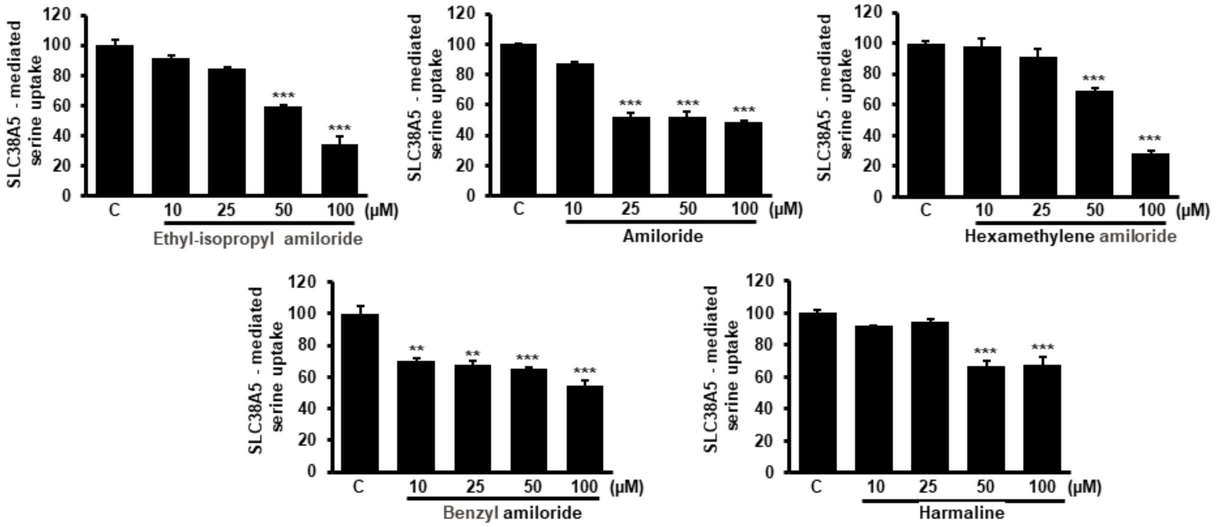

Fig. S5. Inhibition of SLC38A5-mediated [^{3}H]-serine uptake (1 μM) by amiloride and its derivatives. Serine uptake was measured for 15 min in LiCl-buffer (pH 8.5) containing 5 mM tryptophan. Amiloride and its derivatives were present during uptake at increasing concentrations. Control uptake (C) was taken as 100% in each experiment. Data represent means ± S.E. (n = 6). ANOVA followed by Dunn's test was used to determine the statistical significance among the groups. **, $p<0.01$; ***, $p<0.001$.

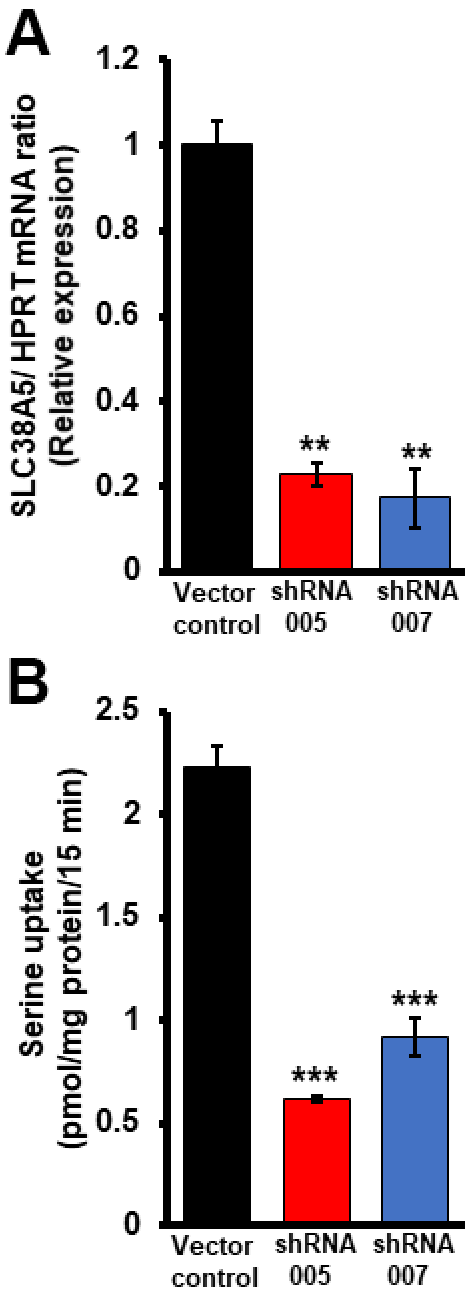

Fig. S6. SLC38A5 mRNA levels (A) and SLC38A5-transport activity (Li^+-stimulatable serine uptake in the presence of tryptophan) (B) in control and shRNA-transfected cells. **, p<0.01compared to vector control; ***, p<0.001 compared to vector control.

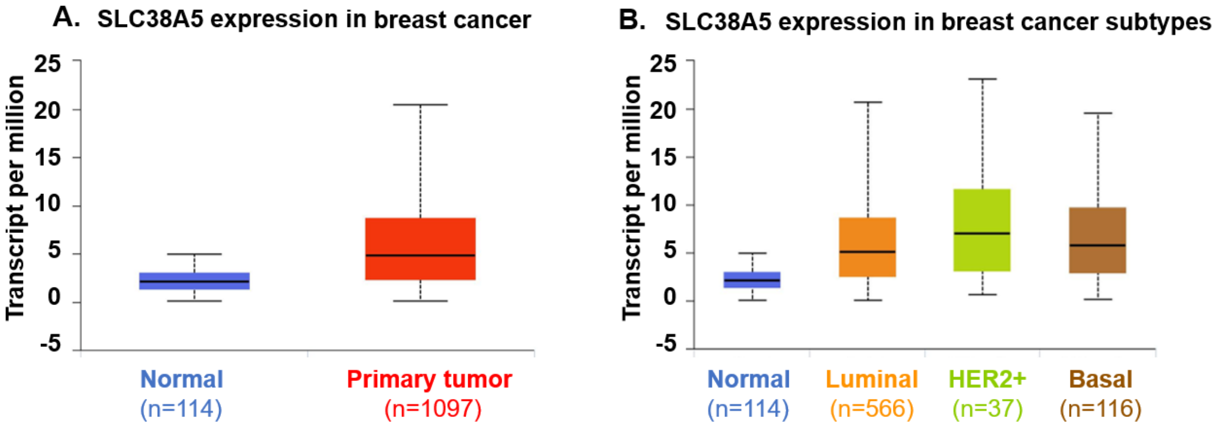

Comparision	Statistical Significance
Normal vs Primary	1.6E-12

Comparision	Statistical Significance
Normal vs Luminal	<E-12
Normal vs HER2+	2.95E-07
Normal vs Basal	3.87E-07

Fig. S7. Expression pattern for SLC38A5 in breast cancer. (A) The TCGA database was used to analyze the expression level of SLC38A5 mRNA in primary breast tumor tissues and in normal mammary gland tissue. (B) The same database was used to analyze the expression pattern for SLC38A5 mRNA in normal mammary gland and in breast cancer of the three major breast cancer subtypes. The numbers in parentheses represent the number of cases in each category.

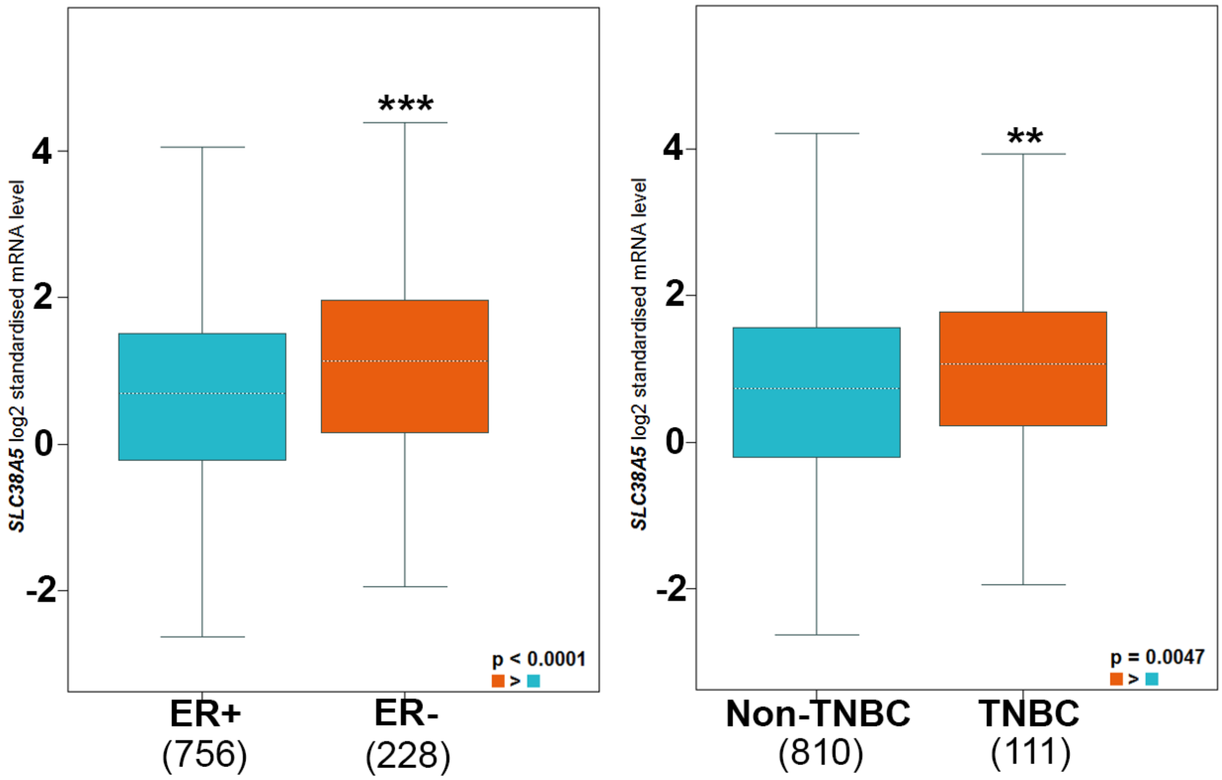

Fig. S8. Relative expression of SLC38A5 mRNA in ER-positive (ER+) vs ER-negative (ER-) breast cancer, and in TNBC versus non-TNBC. The information in the TCGA database was accessed and reformatted. The numbers in parentheses denote the number of cases in each category.

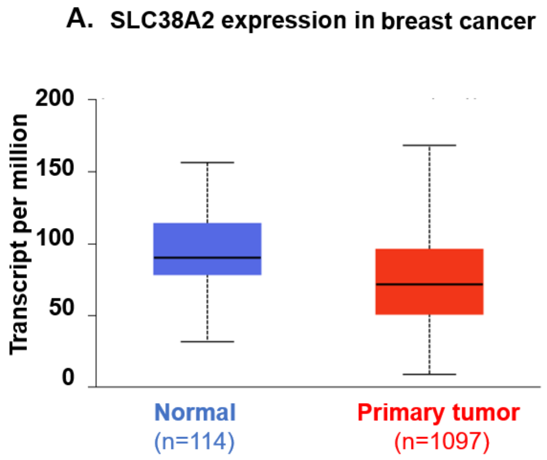

Comparision	Statistical Significance
Normal vs Primary	1.1E-6

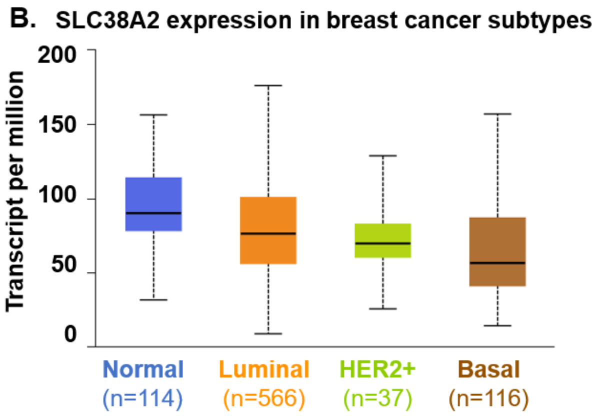

Comparision	Statistical Significance
Normal vs Luminal	9.4E-04
Normal vs HER2+	5.2E-03
Normal vs Basal	1.5E-08

Fig. S9. (A) Expression pattern for SLC38A2 mRNA in primary breast cancer tissues and in non-involved normal tissues from the mammary gland. (B) Expression pattern of SLC38A5 in normal mammary gland and in the three major subtypes of breast cancer. The numbers in parentheses represent the number of cases in each category.
